# Umbravirus-like RNA viruses are capable of independent systemic plant infection in the absence of encoded movement proteins

**DOI:** 10.1371/journal.pbio.3002600

**Published:** 2024-04-25

**Authors:** Xiaobao Ying, Sayanta Bera, Jinyuan Liu, Roberto Toscano-Morales, Chanyong Jang, Stephen Yang, Jovia Ho, Anne E. Simon

**Affiliations:** 1 Department of Cell Biology and Molecular Genetics, University of Maryland, College Park, Maryland, United States of America; 2 Silvec Biologics, Inc., Gaithersburg, Maryland, United States of America; The Sainsbury Laboratory, UNITED KINGDOM

## Abstract

The signature feature of all plant viruses is the encoding of movement proteins (MPs) that supports the movement of the viral genome into adjacent cells and through the vascular system. The recent discovery of umbravirus-like viruses (ULVs), some of which only encode replication-associated proteins, suggested that they, as with umbraviruses that lack encoded capsid proteins (CPs) and silencing suppressors, would require association with a helper virus to complete an infection cycle. We examined the infection properties of 2 ULVs: citrus yellow vein associated virus 1 (CY1), which only encodes replication proteins, and closely related CY2 from hemp, which encodes an additional protein (ORF5_CY2_) that was assumed to be an MP. We report that both CY1 and CY2 can independently infect the model plant *Nicotiana benthamiana* in a phloem-limited fashion when delivered by agroinfiltration. Unlike encoded MPs, ORF5_CY2_ was dispensable for infection of CY2, but was associated with faster symptom development. Examination of ORF5_CY2_ revealed features more similar to luteoviruses/poleroviruses/sobemovirus CPs than to 30K class MPs, which all share a similar single jelly-roll domain. In addition, only CY2-infected plants contained virus-like particles (VLPs) associated with CY2 RNA and ORF5_CY2_. CY1 RNA and a defective (D)-RNA that arises during infection interacted with host protein phloem protein 2 (PP2) in vitro and in vivo, and formed a high molecular weight complex with sap proteins in vitro that was partially resistant to RNase treatment. When CY1 was used as a virus-induced gene silencing (VIGS) vector to target PP2 transcripts, CY1 accumulation was reduced in systemic leaves, supporting the usage of PP2 for systemic movement. ULVs are therefore the first plant viruses encoding replication and CPs but no MPs, and whose systemic movement relies on a host MP. This explains the lack of discernable helper viruses in many ULV-infected plants and evokes comparisons with the initial viruses transferred into plants that must have similarly required host proteins for movement.

## Abstract 2

The spread of viral genomes into adjacent cells and the plant vascular system is enabled by viral movement proteins. This study shows that umbravirus-like RNA viruses can spread systemically without encoding any, through the interaction of viral replication and capsid proteins with host protein PHLOEM PROTEIN 2.

## Introduction

To be a functional plus-strand (+)RNA plant virus, genomes must be translated upon cell entry, replicated, and nascent genomes transported through intercellular plasmodesmata (PD) connections into adjacent cells as ribonucleoproteins (RNP) or after packaging into virions. After cell-to-cell movement, RNPs or virions enter the vascular system for long-distance movement through phloem sieve elements, and then exit and continue cell-to-cell spread. Some viruses are confined to the vascular system and only replicate within phloem-associated nucleated cells. Regardless of tropism, newly synthesized (+)RNA genomes require encapsidation into virions for plant-to-plant transmission either by vectors or by mechanical means [1]. To accomplish their infection cycle, plant (+)RNA viruses are expected to code for: (i) one or more replication proteins, including the core RNA-dependent RNA polymerase (RdRp); (ii) one or more movement proteins (MPs) to interact with PD components for cell-to-cell and/or long-distance movement; (iii) RNA silencing suppressor(s) to suppress the plant’s innate defense system against viruses; and (iv) capsid proteins (CPs) for encapsidation of the genomic (g)RNA(s) [1].

Plant viruses likely originated from viruses that were transferred into the plant by plant-feeding nematodes or arthropods, and which at minimum would have coded for an RdRp and at least 1 CP [2]. To achieve a systemic infection in plants, ancient viruses would have needed to take advantage of a plant’s intercellular communication pathway that currently transports endogenous RNAs through the vascular system to other organs in poorly defined RNP complexes [3–6]. Modern plant virus MPs may have evolved from host MPs, as implied by the MP of red clover necrotic mosaic virus having a pumpkin RNA-binding paralog that can mediate transport of RNA through PD [7], or by duplication of a CP open reading frame (ORF) that then evolved MP functionality, as was recently proposed based on the high structural similarity of some MPs and CPs in their core domain [8]. Since encoding an MP is the key defining feature of modern plant viruses, these virus-encoded MPs must have afforded an advantage in transporting the viral genome over endogenous MPs. Of all modern-day infectious RNAs, only circular noncoding viroids are thought to still use host MPs. For example, hop stunt viroid (HSVd) uses phloem protein 2 (PP2), an enigmatic, dimeric, chitin-binding lectin encoded by a large gene family that is highly abundant in phloem sap and is considered to be a nonspecific RNA-binding protein [9]. PP2 has also been implicated in plant virus transmission, as purified PP2 mixed with virions and fed to aphids can increase the virus transmission rate as well as virion stability in vitro [10].

Unlike host MPs, viral MPs have been extensively studied [11,12]. Viral MPs are nonspecific RNA-binding proteins that assist in the transport of large RNP complexes while simultaneously protecting viral RNAs from degradation by host defenses [11]. Viral MPs, which range in size from 8 kDa to 58 kDa, associate with the cytoskeleton and ER networks to move viral RNA complexes intracellularly to the PD, where most can increase the PD size exclusion limit (SEL) in a process known as “gating” [11–14]. At the PD, MPs mediate gating either without extensive modification of PD structure or by forming homomeric tubules through which virions transit between cells [11,12]. The most extensive class of MPs are the 30K MPs, named after the tobacco mosaic virus 30 kDa MP, which have a single jelly roll central core domain comprising 7 or 8 β-strands that contains a nearly invariant aspartic acid residue that is critical for MP function [8,15]. The N-terminal portion of 30K MPs is involved in PD targeting [16,17], while the C-terminal region can interact with CPs and/or can support long-distance movement through the vascular system [18–20]. Alterations in conserved MP residues restrict a virus to initially infected cells [21,22], and viruses with multiple MPs or chimeric viruses with heterologous MPs have an extended host range [23–26], supporting the critical role of encoded MPs in the infection cycle of plant viruses. Additionally, CPs can regulate movement of viral gRNAs by increasing the RNA-binding affinity of 30K MPs [13,27] and can also be indispensable for reaching vascular tissues without a requirement for virion formation [28–31].

Umbraviruses (family Tombusviridae) are unusual in that they do not encode CPs or silencing suppressors. Rather, umbraviruses rely on a helper virus from the luteovirus/enamovirus/polerovirus genera for silencing suppression and for supplying CP for gRNA encapsidation [32]. Umbraviruses have a 4.0 to 4.5 kb monopartite (+)RNA genome containing 4 ORFs [33–36]. The two 5′ proximal ORFs (ORF1 and ORF2) encode replication-required proteins including the RdRp, which is produced by −1 ribosomal frameshifting just upstream of the ORF1 termination codon [37]. The other 2 ORFs, which are overlapping and located downstream of an intergenic region, encode a long-distance MP (ORF3) and a 30K class cell-to-cell MP (ORF4), both of which are translated from a bicistronic subgenomic (sg)RNA [38].

**Fig 1:**
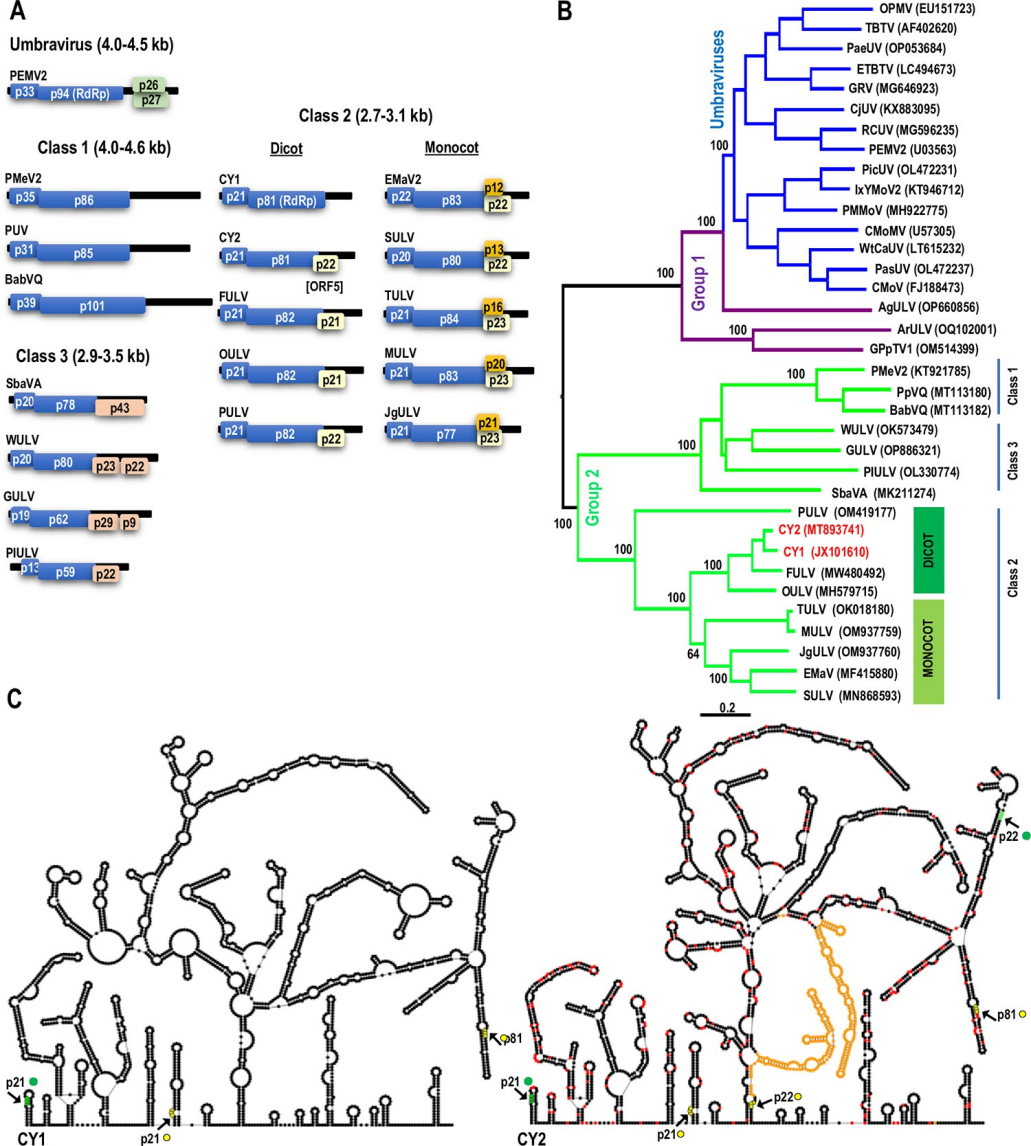
CY1 and CY2 are closely related Group 2/Class 2 ULVs. **(A)** Genome organization of ULVs. Group 2/Class 1 members only encode 2 replication-required proteins and have extensive 3′ UTRs. Group 2/Class 2 members that infect dicots (except CY1) have an additional ORF (ORF5) that overlaps with the end of the RdRp ORF in the −1 frame. Monocot-infecting Class 2 members have an extra embedded ORF of different lengths. Group 2/Class 3 members have at least 1 additional ORF that are unrelated to each other and ORF5. **(B)** Maximum-likelihood phylogenetic tree based on RdRp nucleotide sequences. Branch numbers indicate bootstrap support in percentage out of 1,000 replicates. The scale bar denotes nucleotide substitutions per site. The tree is mid-point rooted. Dicot-infecting and monocot-infecting Class 2 ULVs separate into different clades with the exception of PULV, which is closer to an ancestral viral molecule that possibly gave rise to all Class 2 ULVs. **(C)** Left, secondary structure of CY1 [39]. CY2 shares a similar overall secondary structure and contains 2 large segments (in orange) missing in CY1. Green dots denote start codons and yellow dots denote stop codons. Nucleotide differences between CY2 and CY1 are denoted with red dots. ORF, open reading frame; PULV, parsley umbra-like virus; RdRp, RNA-dependent RNA polymerase; ULV, umbravirus-like virus.

A number of viruses have been recently identified that encode an umbravirus-related RdRp that is generated by −1 ribosomal frameshifting, and have umbravirus-related 3′ terminal RNA structures, but do not encode umbravirus-like MPs (Figs 1A and S1) [39,40]. Based on phylogenetic analyses, umbravirus-like viruses (ULVs) have been divided into 2 groups. Group 1 is a catch-all grouping of viruses that are more related to umbraviruses than Group 2 (Figs 1B and S1A). Group 2 members subdivide into 3 classes (Fig 1A and 1B): Class 1 members only contain ORFs 1 and 2 while Class 2 members have an additional ORF (ORF5) that overlaps with the end of the RdRp ORF [41,42]. The one exception in Class 2 is citrus yellow vein associated virus (CY1, also known as CYVaV), which no longer encodes ORF5 due to 2 large deletions and other alterations (Fig 1C) [40]. CY2, a close relative of CY1 that is found in hemp [43], still contains ORF5. Class 2 members that infect monocots have an additional ORF of varying lengths in the −2 frame that is nearly completely embedded within ORF5. All Class 3 members have their own unique additional ORF(s) that are unrelated to ORF5 [39].

Surprisingly, several Group 2 ULVs were reported to exist in plants in the apparent absence of a helper virus [40–42,44]. These include members of Class 1 that infect papaya and babaco trees throughout Ecuador, and Class 2 opuntia umbra-like virus (OULV) found in opuntia plants. Likewise, CY1, which was discovered only once in Southern California limequat trees with yellow vein symptoms in the 1950’s, is currently present without a discernable helper virus in citrus trees that were infected with grafts from the original source material [40]. These reports of ULVs infecting plants in the apparent absence of a helper virus, despite some only encoding proteins that are known to be involved in replication in related members of the Tombusviridae [45,46], suggest that some ULVs exist autonomously in plants without encoding an MP.

To evaluate if ULVs can infect plants in the absence of an encoded MP and/or helper virus, we generated full-length clones of CY1 and CY2 and examined their abilities to systemically infect the model plant *Nicotiana benthamiana*. We report that both CY1 and CY2 were capable of independent systemic infection in a phloem-limited fashion when delivered by agroinfiltration. ORF5 was dispensable for infection of CY2, but was associated with faster symptom development. Unexpectedly, the CY2 ORF5 protein (ORF5_CY2_) has characteristics more similar to luteoviruses/poleroviruses/sobemovirus CPs than to 30K MPs, which all share a similar single jelly-roll domain. In addition, CY2-infected plants contained small virus-like particles (VLPs), suggesting that CY2 uses ORF5 as a CP. As with viroid HSVd, CY1 interacted with phloem protein PP2 in sap, and formed a high molecular weight complex with sap proteins in vitro that was partially resistant to RNase treatment. When CY1 was used as a virus-induced gene silencing (VIGS) vector to reduce PP2 transcripts, CY1 accumulation was also reduced in systemic leaves, supporting the possibility that PP2 is the MP for CY1 and CY2. ULVs thus represent the first plant viruses, to our knowledge, whose systemic movement relies on a host MP, evoking comparisons to ancient viruses that must have similarly relied on host proteins for movement.

## Results

### CY1 systemically infects *N*. *benthamiana* and Mexican lime in the absence of a helper virus

**Fig 2:**
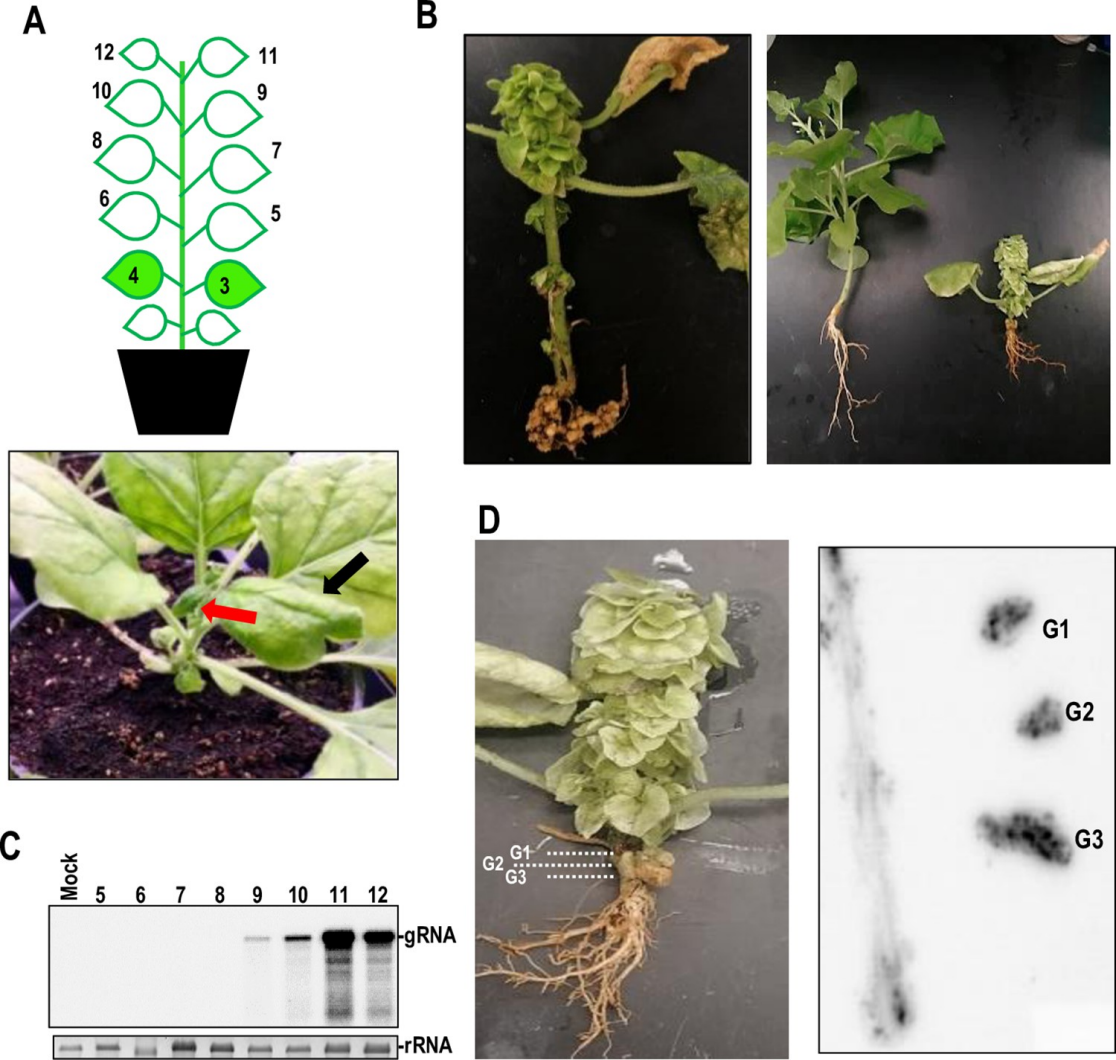
CY1 systemically infects *N*. *benthamiana* in the absence of a helper virus. **(A)** Leaves 3 and 4 were agroinfiltrated at the 4-leaf stage with a Ti plasmid expressing full-length CY1 transcripts. Initial heart-shaped leaf (leaf 9; black arrow) was visible at 21 dpi. Subsequent emerging leaves were small and curled down at the margins (red arrow). **(B)** Typical phenotype of infected plants at 50 dpi. Multiple leaves emerged at each node and roots contained multiple galls. Infected plants were stunted compared with uninfected plants of the same age (right panel). Infected plants senesced within 3 to 4 months post-infiltration. **(C)** Detection of CY1 in systemic upper leaves by northern blot at 19 dpi. Total RNA was extracted from specified leaves (leaf numbering shown in **A**) and subjected to northern blot analysis. Ethidium-bromide stained rRNA was used as a loading control. **(D)** Stem and 3 sections of the root gall were excised from a 50 dpi plant (shown in left panel) and pressed onto nitrocellulose, which was probed for CY1 (right panel). G1-3, galls. dpi, days post-infiltration.

We previously determined that transcripts synthesized from a full-length clone of CY1 replicated to similar levels as umbravirus pea enation mosaic virus 2 (PEMV2) in *Arabidopsis thaliana* protoplasts [39]. This result was unexpected as CY1 only contains the umbravirus replication-associated ORFs and thus lacks umbravirus ORF3. The ORF3 protein, in addition to supporting long-distance movement, also functions to inhibit nonsense-mediated decay, which is necessary for efficient replication of PEMV2 in protoplasts [47]. The lack of a detectable helper virus in CY1-infected citrus trees [40] suggested that CY1 is also capable of independent systemic infection despite the lack of an encoded MP. To determine the validity of this hypothesis, *N*. *benthamiana* plants were agroinfiltrated with CY1 and examined daily for symptom development. Of the initially infiltrated plants, 10% developed an abnormal heart-shaped leaf at 21 days post-infiltration (dpi), and subsequent leaves emerging at the apex were uniformly small and narrow, with severely curled margins (Fig 2A, lower panel, and 2B). Clusters of small leaves began emerging from nodes at about 30 dpi, and plants also were substantially stunted (Fig 2B and 2D). Examination of roots at late stages of infection revealed the presence of 1 or more large galls that were absent from uninfected plants. Total RNA extracted from systemic leaves of newly infiltrated seedlings at 19 dpi and hybridized with CY1-specific probes revealed the presence of CY1 gRNA, with levels increasing in leaves near the apex (Fig 2C). CY1 gRNA was also detected in stems and root galls at 50 dpi (Fig 2D).

To determine if CY1 can also independently infect citrus, Mexican lime was inoculated with CY1 either by direct agroinfiltration or by using dodder to transfer the virus from infected *N*. *benthamiana* (S2A Fig). Total RNA that was extracted from selected plants 12 or 15 months later, respectively, contained CY1 gRNA that was absent from uninfected plants (S2B Fig, right panel). Unlike *N*. *benthamiana*, citrus infected with CY1 did not show any discernable symptoms (S2B Fig, left panel). These results strongly suggest that CY1 is capable of independent systemic infection of plants despite the lack of encoded MPs.

### CY1 is phloem-limited in infected stems and roots

**Fig 3:**
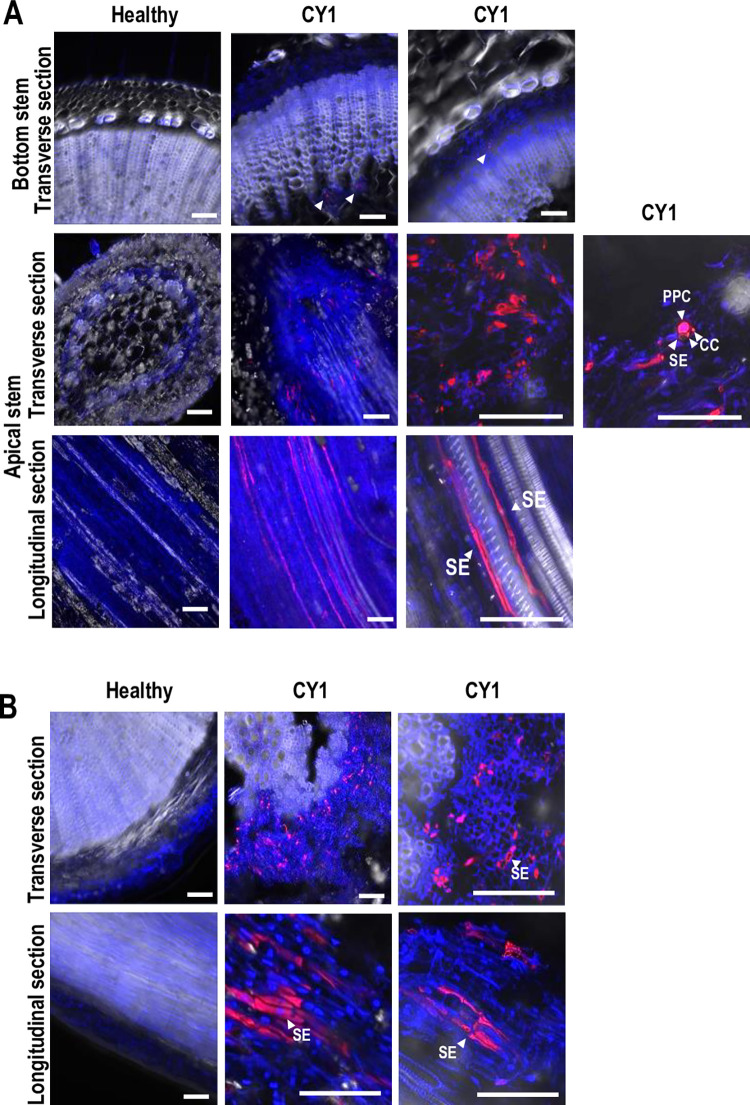
Systemic infection of CY1 is limited to vascular tissue. FISH analysis showing accumulation of CY1 RNA in stem and root sections of infected *N*. *benthamiana* at 60 dpi. **(A)** Transverse section of the bottom stem, and transverse and longitudinal sections of apical stem of healthy and CY1-infected plants. Cy3-labeled CY1 probe produced the fluorescent signal. Arrowheads denote signals in specific phloem locations. DAPI-stained DNA and xylem tissue fluoresce blue. All sections were stained with ProLong Gold Antifade Mountant (Invitrogen, USA). Bars = 60 μm. PPC, phloem parenchyma cells; SE, sieve tube elements; CC, companion cells. **(B)** Similar analysis of root sections. dpi, days post-infiltration; FISH, fluorescence in situ hybridization.

CY1 distribution in *N*. *benthamiana* was examined by fluorescence in situ hybridization (FISH). CY1 in apical stems was limited to phloem parenchyma cells (PPCs), sieve tubes (SE), and companion cells (CC), while scattered fluorescent signals in lower stems were only found in phloem tissue (Fig 3A). CY1 was also detected in root sections, with signals observed in both immature (nucleated) and mature (enucleated) SE and in adjacent phloem-associated cells (Fig 3B). These results indicate that CY1 movement is bidirectional in *N*. *benthamiana* and strongly suggests that CY1 infection is vascular-limited.

### ORF5_CY2_ is dispensable for replication and movement of CY2

**Fig 4:**
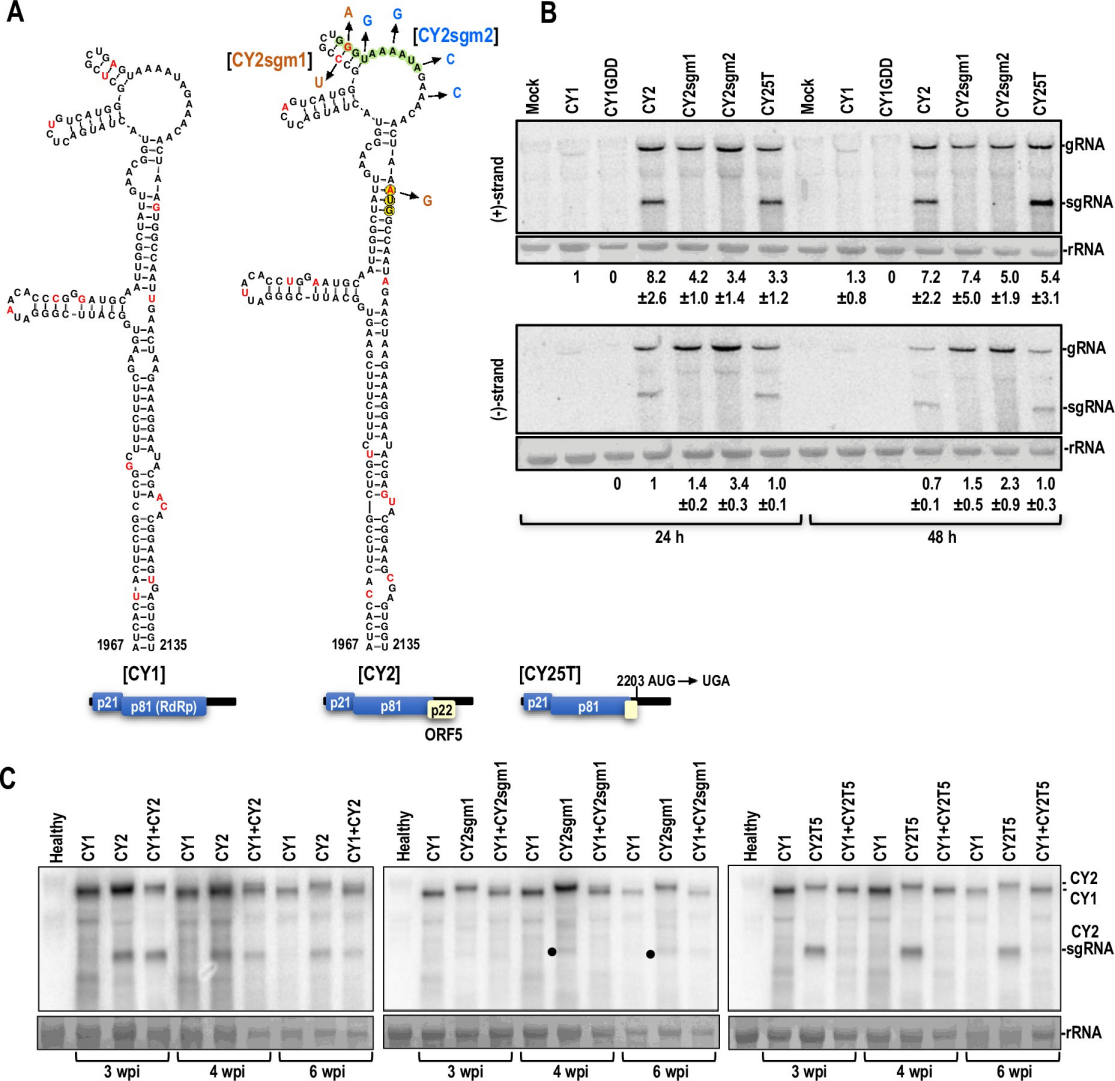
CY2 replicates and moves systemically in the absence of ORF5. **(A)** Secondary structures of a portion of CY1 (left) and the corresponding region of CY2 (right). Sequence shaded light green in CY2 is a “carmovirus consensus sequence” (G_2-3_A/U_5-9_) located at the 5′ ends of carmovirus and umbravirus gRNAs and sgRNAs. Red residues differ between CY1 and CY2. ORF5 initiation codon is circled and shaded yellow. CY2 mutants CY2sgm1 and CY2sgm2 have color-coded alterations in the sgRNA promoter region. CY25T contains a truncated ORF5 protein (33 amino acids remaining). **(B)** Accumulation of (+)- and (-)-strands of CY1, CY2, and CY2 mutants in *Arabidopsis* protoplasts at 24 h and 48 h post-transfection assayed by northern blots. CY1 accumulation was low but detectable. CY1 with an RdRp active site alteration (CY1-GDD) served as a negative control. Mock, no added RNA. Data was obtained from 3 independent experiments. Standard deviation is shown. No significant difference in accumulation of the gRNA was found for CY2 and the CY2 mutants using JMP Pro 16, Student’s *t*, α = 0.05. **(C)** Competition assays between CY1 and WT and mutant CY2. Symptomatic systemic leaves were collected at 3-, 4-, and 6-wpi and total RNA was extracted and subjected to northern analysis. CY1 and CY2 size differences were used to visually assess levels of the different gRNAs. Three independent experiments were conducted with very similar results. Note that while WT CY2 is mainly detected at 3-wpi in co-infiltrated plants, both CY1 and CY2 are present at 4- and 6-wpi (left panel). In contrast, CY1 is mainly detected at all time points in plants co-infiltrated with the ORF5 deficient or defective mutants (middle and right panels). ●, denotes low level band that is not detected in CY2sgm1 protoplasts infections that is near the size of the sgRNA (831 nt) and is also the size of a defective (D)-RNA (921 nt) that commonly arises in CY1/CY2 infections. ORF, open reading frame; RdRp, RNA-dependent RNA polymerase; wpi, weeks post-infiltration; WT, wild type.

Since the evolutionary loss of ORF5 did not preclude CY1 systemic infection, we next investigated CY2 infection in the presence and absence of its presumed MP, ORF5_CY2_. Unlike CY1, CY2 contains a perfect carmovirus consensus sequence (CCS; GGGUAAAUA) just upstream of the ORF5_CY2_ initiation codon (Fig 4A, in green). CCSs are present at the 5′ ends of gRNAs and sgRNAs in carmoviruses, umbraviruses, and members of some other genera within the Tombusviridae [38,48,49], and sgRNA promoters are located just upstream of the CCS [50]. Two sets of mutations were generated within and upstream of the CCS to engineer CY2 variants that were incapable of generating sgRNA, and thus should preclude synthesis of ORF5_CY2_ (Fig 4A). CY2sgm1 contains mutations that altered the CCS central guanylate and an upstream residue (to maintain local RNA structure) and also eliminated the ORF5_CY2_ initiation codon; CY2sgm2 contains 3 alterations in the CCS and a fourth alteration in a nearby downstream residue. Since there was a possibility that ORF5_CY2_ could still be synthesized by internal initiation, similar to carmovirus turnip crinkle virus CP [51], an additional construct was generated (CY2T5) with a single alteration that introduced a UGA stop codon at position 2203, which would produce a truncated, 33 amino acid ORF5_CY2_. None of the alterations affected the sequence of the RdRp or the presumptive secondary structure of the gRNA.

*Arabidopsis* protoplasts were transfected with CY1, CY1_GDD_ (CY1 with an altered RdRp active site), CY2, CY2sgm1, CY2sgm2, and CY2T5. CY2 (+)- and (-)-strands accumulated to substantially higher levels than CY1 at 24 and 48 hours post-transfection (hpi) (Fig 4B). As expected, only protoplasts infected with CY2 and CY2T5 were associated with a prominent sgRNA of the expected size (Fig 4B). No significant difference was found for the accumulation of CY2 and the CY2 ORF5 mutants (+)- or (-)-strands in protoplasts. The reason for the increased accumulation of CY2 gRNA compared with CY1 is not known; however, these data show that CY2sgm1 and CY2sgm2 do not detectably express the CY2 sgRNA and that none of the CY2 variants have impaired gRNA replication.

To determine if systemic infection requires ORF5 expression, *N*. *benthamiana* plants were infiltrated with the same constructs. Plants infiltrated with wild-type (WT) CY2 produced symptoms similar to CY1; however, the heart-shaped leaf appeared at 2 weeks post-infiltration (wpi) in the majority of plants, which was 1 week earlier than found for CY1-infiltrated plants (Table 1). In addition, CY2-infected plants exhibited greater stunting, likely a consequence of earlier symptom development. All 3 ORF5_CY2_-defective mutants produced similar symptoms as CY2, but timing of symptoms and degree of stunting was more similar to that of CY1 (Table 1). To determine if there was a fitness cost associated with the absence of ORF5, competition assays were performed between CY1 and WT or mutant CY2. In plants co-infiltrated with CY1 and CY2, CY2-sized gRNA was more prominent at 3 wpi (Fig 4C, left panel). At 4 and 6 wpi, however, both CY2- and CY1-sized gRNAs were discernable, suggesting that CY1 and CY2 had similar fitness despite CY1’s lack of ORF5 and slower initial infection. In contrast, CY1 accumulation was clearly favored at 4- and 6-wpi in plants co-infiltrated with either CY2sgm1 or CY25T (Fig 4C, middle and right panels). This result suggests that CY1 is more fit than CY2 ORF5 mutants, which may reflect adaption of CY1 within long-lived citrus in the absence of ORF5. Altogether, these results indicate that ORF5_CY2_ is not required for systemic infection of CY2 or CY1, but rather plays a supportive role that decreases the timing to initial symptoms.

**Table 1:** Percentage of *N*. *benthamiana* plants showing symptoms at different times post-infiltration of CY1, CY2, and CY2 ORF5 mutants.

Virus	# of plants infiltrated	Plants with symptoms (wpi)			
		2	3	4	6
**CY1**	**50**	**6% b**	**48% b**	**64% ab**	**64% b**
**CY2**	**48**	**59% a**	**87% a**	**87% a**	**87% a**
**CY2T5**	**45**	**6% b**	**33% bc**	**56% b**	**63% b**
**CY2sgm1**	**45**	**2% b**	**30% c**	**59% b**	**64% b**
**CY2sgm2**	**48**	**5% b**	**34% bc**	**64% ab**	**64% b**

These results were pooled from three independent experiments. For statistical analysis, independent experiments were considered replicates to calculate means that were further subjected to Student’s *t*, α = 0.05, for pairwise comparisons. Means with the same letter(s) are not significantly different within a column. wpi, weeks-post infiltration. Source data can be found in S1 Data.

To determine if ORF5_CY2_ alters the vascular-limited tropism of CY1 in infected plants, transverse sections of apical stems, bottom stems, petioles, and roots from a healthy plant as well as CY1-, CY2-, and CY2sgm1-infected plants were examined by confocal microscopy (S3 Fig). Fluorescent signal distribution was similar for all viruses and variants, which remained limited to phloem-associated cells. These results suggest that the presence of ORF5_CY2_ does not affect tissue tropism of either CY2 or CY1 in infected plants.

### ORF5_CY2_ also accelerates infection of CY1

Enhanced accumulation of CY1 in plants co-infiltrated with CY2sgm1 and CY25T suggested that CY1 has adapted to compensate for the loss of ORF5. To determine if CY1 infection can be accelerated by ORF5_CY2_, transgenic *N*. *benthamiana* plants expressing ORF5_CY2_ under the cauliflower mosaic virus (CaMV) 35S promoter (N.b.-ORF5_CY2_) were generated. Transgenic *N*. *benthamiana* expressing OULV ORF5 (N.b.-ORF5_OULV_) were also generated in case CY1 was silenced by the more closely related CY2 ORF5 sequence, most of which is still present in CY1. Transgenic plants, which were confirmed to be expressing the respective ORF5 proteins (S4 Fig), along with WT *N*. *benthamiana* were infiltrated with CY1 and timing to first symptoms determined (Table 2). At 2 wpi, no WT plants infiltrated with CY1 developed symptoms, whereas 22% to 48% of transgenic plants displayed CY1-specific symptoms. At 3 wpi, 32% of WT plants were symptomatic compared with 61% to 86% of infected transgenic plants. Finally, at 4 wpi, 59% of WT *N*. *benthamiana* plants were symptomatic compared with 78% to 90% of transgenic plants. These data strongly suggest that CY1 infects more rapidly in the presence of ORF5 proteins. Altogether, our results indicate that Class 2 ULVs can systemically infect the vascular system of plants while encoding only umbravirus-related replication proteins and that the ORF5 protein functions to expedite the initial infection.

**Table 2:** CY1 infects more rapidly in transgenic plants expressing CY2 or OULV ORF5.

Plant lines	# of plants infiltrated	Plants with symptoms (wpi)		
		2	3	4
**WT N.b.**	**22**	**0% b**	**31% b**	**54% b**
**N.b.-ORF5**_**CY2**_ **#1**	**23**	**20% ab**	**61% ab**	**76% ab**
**N.b.-ORF5**_**CY2**_ **#2**	**20**	**29% ab**	**79% a**	**83% ab**
**N.b.-ORF5**_**OULV**_ **#1**	**24**	**35% ab**	**80% a**	**87% ab**
**N.b.-ORF5**_**OULV**_ **#2**	**21**	**45% a**	**85% a**	**89% a**

These results were from three independent experiments. For statistical analysis, independent experiments were considered replicates to calculate means that were further subjected to Student’s *t*, α = 0.1, for pairwise comparisons. Means with the same letter(s) are not significantly different within a column. wpi, weeks-post infiltration; N.b., *Nicotiana benthamiana*. Source data can be found in S1 Data.

### ORF5_CY2_ cannot substitute for tobacco mosaic virus MP or CP

Earlier symptoms in CY2-infected *N*. *benthamiana* (Table 1) and CY1-infected transgenic *N*. *benthamiana* (Table 2) compared with CY1 and CY2 mutant-infected plants suggested that ORF5_CY2_ might be functioning as an MP. However, the dispensability of ORF5 for movement suggested that if ORF5 protein is an MP, it is not a canonical MP. To further examine if ORF5_CY2_ is a viral MP, we investigated the ability of ORF5_CY2_ to substitute for the TMV MP or CP in a GFP-expressing TMV vector (S5 Fig). Two different TMV vectors were designed containing either a truncated MP or a deleted CP, both of which were incapable of systemic movement in infiltrated *N*. *benthamiana*. Replacing either the TMV MP or CP with ORF5_CY2_ did not restore systemic movement (S5 Fig), leaving the possibility that ORF5_CY2_ is not functioning as a canonical MP.

### ORF5_CY2_ shares structural similarity with 30K MPs and luteovirus/polarovirus/sobemovirus CPs

**Fig 5:**
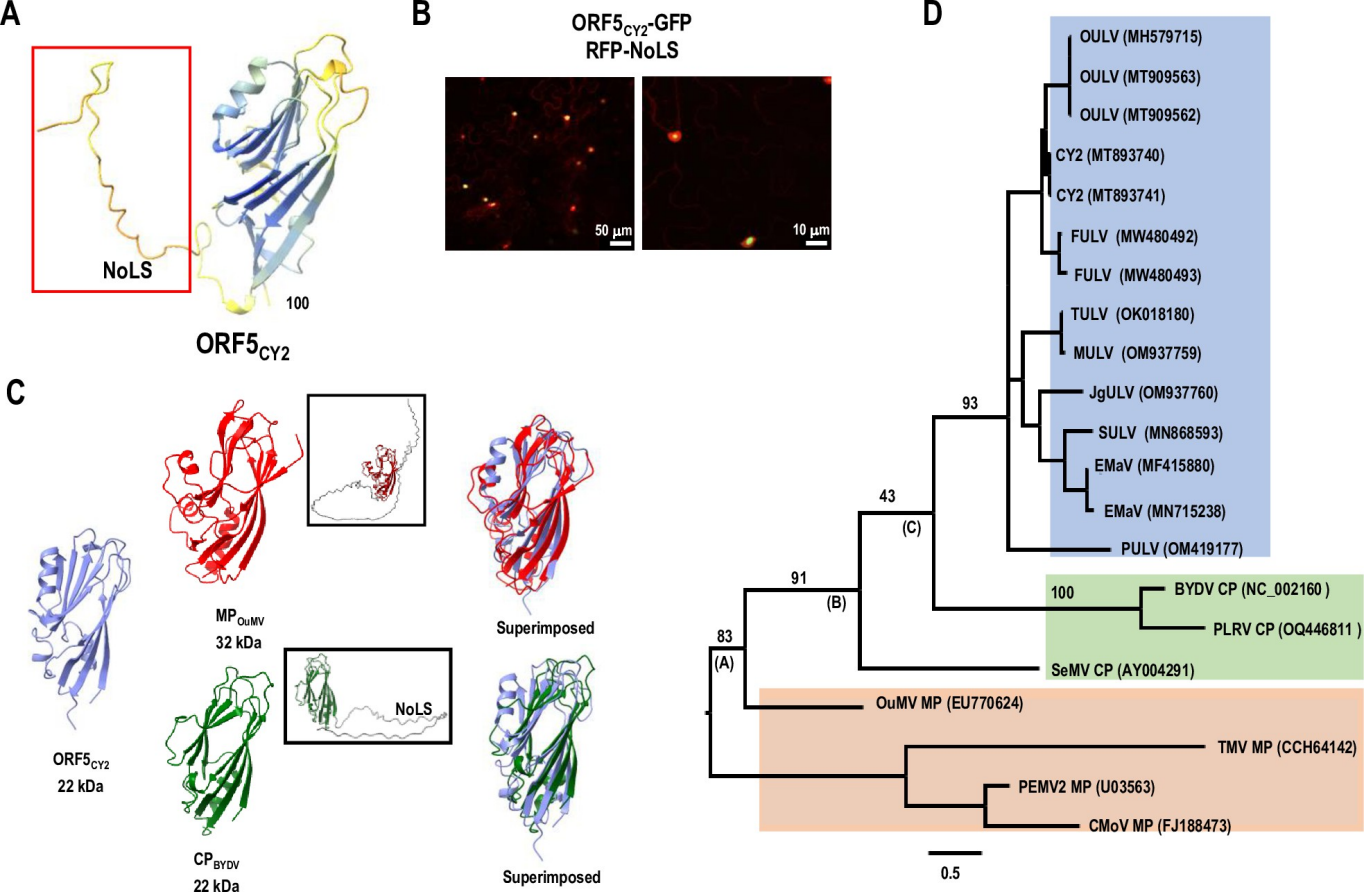
ORF5_CY2_ is structurally similar to 30K MPs and luteovirus/polerovirus/sobemovirus CPs. **(A)** Structure modeling of full-length ORF5_CY2_ using Alphafold2. Color coding from blue to red indicates high to low structural confidence, respectively. Unstructured N-terminal region contains a predicted nucleolar localization signal (NoLS). **(B)** Overlay images of ORF5_CY2_-GFP and RFP-NoLS in *N*. *benthamiana*. Leaves were co-infiltrated with Agrobacteria expressing ORF5_CY2_-GFP and RFP-NoLS from CaMV 35S promoters. Infiltrated leaves were collected at 2 dpi and subjected to confocal microscopy. **(C)** Single jelly-roll domains of ORF5_CY2_, OuMV MP (MP_OuMV_), and BYDV P3 CP (CP_BYDV_). Full-length structures of MP_OuMV_ and CP_BYDV_ are boxed. CP_BYDV_ also contains an N-terminal NoLS. **(D)** Maximum-likelihood phylogenetic tree based on amino acid sequences of Class 2 ORF5 (blue), representative 30K MPs (red), and SeMV (sobemovirus), BYDV (luteovirus), and PLRV (polerovirus) CPs (green). Branch numbers indicate bootstrap support in percentage out of 1,000 replicates. The letters beneath the branch numbers indicate the node names. The scale bar denotes amino acid substitutions per site. The tree is mid-point rooted. Protein sequences were aligned through PROMALS3D and subjected to phylogenetic analysis in MEGA X. BYDV, barley yellow dwarf virus; CP, capsid protein; dpi, days post-infiltration; MP, movement protein; ORF, open reading frame; OuMV, ourmia melon virus; PLRV, potato leafroll virus; SeMV, sesbania mosaic virus.

To further investigate the function of the ORF5 protein, a structural model of ORF5_CY2_ was generated using Alphafold2, which predicted with high confidence the presence of a jelly-roll domain containing 8 β-strands (Fig 5A). In addition, the N-terminal region of ORF5_CY2_ (and all other Class 2 ORF5 proteins) contains an unstructured, arginine (R)-rich sequence that was predicted to contain a nucleolar localization signal (NoLS) (S6A Fig). To support the presence of a functional ORF5 NoLS, *N*. *benthamiana* leaves were co-infiltrated with a plasmid expressing an ORF5_CY2_-GFP fusion protein and a second plasmid expressing RFP-NoLS. Infiltrated leaf samples collected at 2 dpi and observed under a confocal microscope showed that nearly all red nucleoli that were sequestering RFP-NLS also contained green/yellow dots indicating the co-localization of GFP-tagged ORF5_CY2_ (Fig 5B). These data support the presence of a functional NoLS in the N-terminal region in ORF5_CY2_.

Three-dimensional structural models were recently generated for members of the 30K MP superfamily and were shown with high confidence to contain a conserved central region containing a similar 7 or 8 β-strand jelly-roll domain that also resembles the jelly-roll topologies of some CPs [8]. As CPs can assist in viral RNA movement, a bioinformatic approach was taken to manually curate the relatedness of the structural model for ORF5_CY2_ with representative 30K MPs and CPs. The MP most closely resembling ORF5_CY2_ was the 30K class MP of ourmia melon virus (MP_OuMV_) (Fig 5C, top). Superimposition of the structural models for ORF5_CY2_ and MP_OuMV_ showed good alignment of the jelly-roll domains and partial alignment of α-helices. The structural model for ORF5_CY2_ was then subjected to further analysis using the DALI server, which searches for proteins in the PDB database with a similar structure. The best hit was the 22 kDa CP of luteovirus barley yellow dwarf virus (BYDV), which had a significant Z score of 10 (Fig 5C, bottom) (note that no MPs were identified due to the absence of any high-resolution MP structures in the PDB database). The BYDV CP is the same size as ORF5_CY2_ and also contains an NoLS sequence in its unstructured N-terminal region [52] (S6B Fig).

If ORF5 proteins are 30K class MPs, then they should contain the critical aspartic acid residue within the jelly-roll domain that is required for function. Alignment of ORF5 proteins with selected 30K MPs indicated the absence of this aspartic acid residue (S7A Fig). We also examined the charge distribution across ORF5_CY2_ and other ORF5 proteins, since unlike MPs, CPs are known to have an unequal charge distribution with charged residues concentrated in the N-terminal region [8]. As shown in S7B Fig, ORF5_CY2_ has an unequal charge distribution similar to CPs. To determine if there is a closer evolutionary link between ORF5 proteins and luteovirus/polerovirus/sobemovirus (LPS) genera CPs than with 30K MPs, a maximum-likelihood phylogenetic tree was generated using the aligned amino acid sequences produced in PROMALS3D [53], which uses structural and biophysical characteristics of proteins for alignment. Phylogenetic analyses suggested that ORF5 proteins form a strong clade with LPS CPs (node B), which is a sister clade to MP_OuMV._ The tree also suggested that MP_OuMV_, ORF5 proteins, and LPS CPs share a similar jelly-roll domain derived from a common ancestral protein (Fig 5D, node A), demonstrating an evolutionary relationship between LPS CPs and the MP 30K superfamily, as was recently suggested [8]. Altogether, these results suggest that ORF5 proteins are more related to CPs than MPs.

### ORF5_CY2_-infected plants contain macromolecular structures resembling VLPs

**Fig 6:**
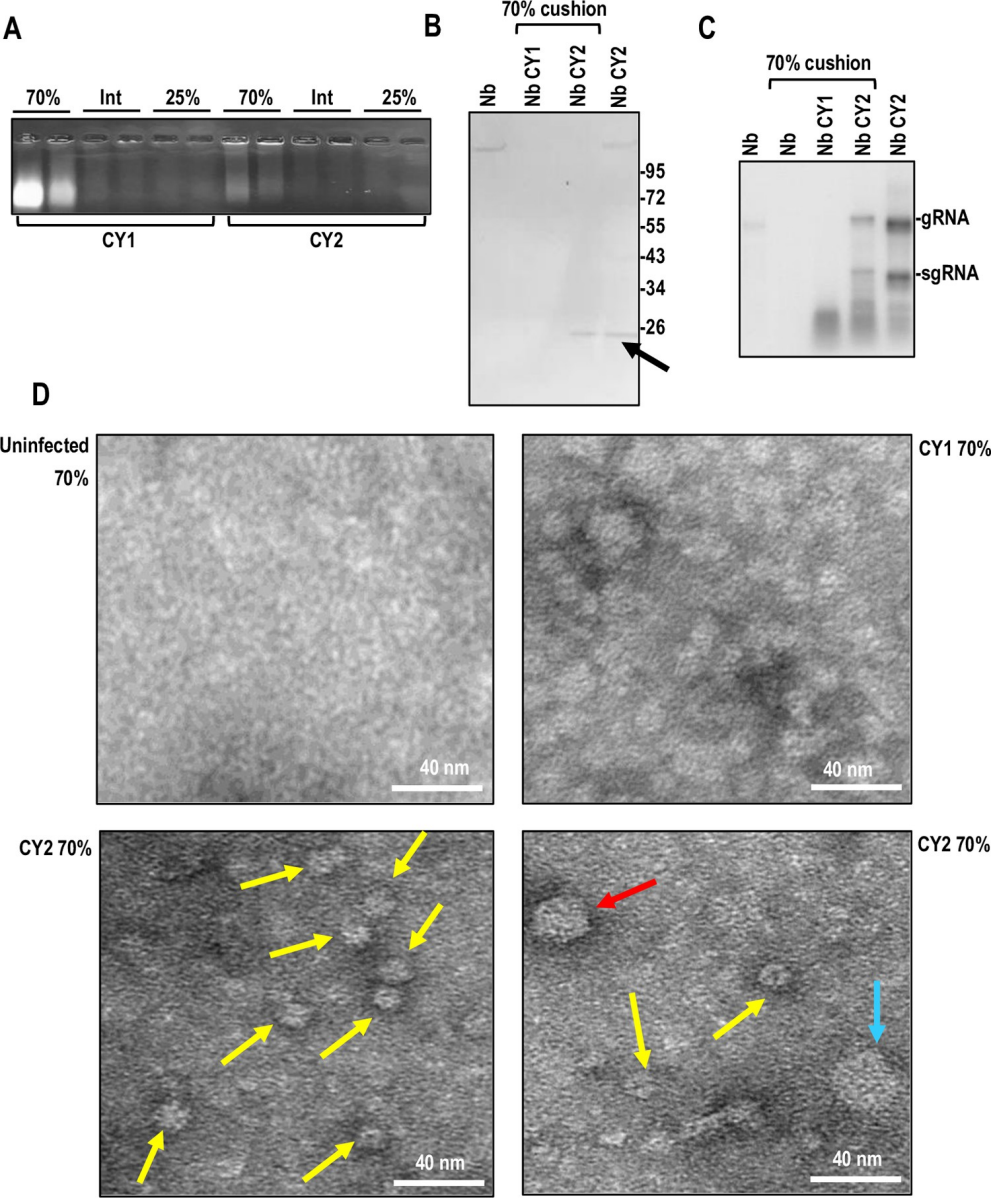
ORF5_CY2_ forms VLPs in infected *N*. *benthamiana*. **(A)** CY1- and CY2-infected plants were subjected to ultracentrifugation to gently purify any VLPs. Material from CY1- and CY2-infected *N*. *benthamiana* in the 70% sucrose cushion, interface (Int), and 25% sucrose cushion was examined following electrophoresis through a 0.8% agarose gel. Nb, *N*. *benthamiana*. **(B)** Western blot detection of ORF5_CY2_. Total proteins from healthy *N*. *benthamiana* and CY2-infected *N*. *benthamiana*, and 70% sucrose fractions of CY1- and CY2-infected *N*. *benthamiana* were separated by 10% PAGE. ORF5_CY2_-specific antibody was used to detect ORF5_CY2_ (arrow). **(C)** Northern blot detection of CY2 gRNA and sgRNA in material from the 70% sucrose fraction of CY2-infected *N*. *benthamiana*. Total RNA from uninfected *N*. *benthamiana* and CY2-infected *N*. *benthamiana* served as negative and positive controls, respectively. **(D)** Samples of 70% sucrose cushions from uninfected *N*. *benthamiana*, CY1-infected *N*. *benthamiana*, and CY2-infected *N*. *benthamiana* were exchanged to PBS buffer and observed by EM. Particles of 3 different sizes were found in CY2-infected *N*. *benthamiana* that were absent from uninfected and CY1-infected *N*. *benthamiana*. Yellow arrows point to CY2-infected *N*. *benthamiana* particles with a diameter of 14 nm that were the most prevalent. Red arrow denotes a 24 nm particle and blue arrow denotes a 35 nm particle. EM, electron microscopy; N b., *N*. *benthamiana*; ORF, open reading frame; VLP, virus-like particle.

Since ORF5 proteins are more closely related to LPS CPs, we wanted to determine if the presence of ORF5 was associated with the presence of VLPs. To examine if CY2-infected plants contained ORF5-associated VLPs, CY1- and CY2-infected *N*. *benthamiana* tissue was subjected to a gentle virion purification protocol [54], and the 70% and 25% sucrose layers and interface were subjected to electrophoresis through 0.8% agarose gels. The 70% sucrose fraction from CY2-infected plants contained high molecular weight material that stained with ethidium bromide and did not migrate into the gels (Fig 6A). Northern blots using CY2-specific probes detected the presence of CY2 gRNA and sgRNA in the 70% sucrose fraction (Fig 6C), and western analysis confirmed the additional presence of ORF5_CY2_ (Fig 6B). High molecular weight material was also present in the 70% sucrose fraction from CY1-infected plants, but this material did not contain detectable full-length CY1 RNA.

Seventy-percent sucrose fraction samples from uninfected *N*. *benthamiana* and CY1- and CY2-infected plants were visualized by electron microscopy (EM). CY2-infected samples contained numerous macromolecular assemblies, the majority of which were uniformly approximately 14 nm in size (Fig 6D, bottom panels). Two additional larger particles were also occasionally found that were 24 nm and 35 nm in diameter (Fig 6D, lower right panel). Samples from CY1-infected plants contained complexes of irregular size and shape without the sharp contrast boundary observed for the particles from CY2-infected plants. None of the CY2 or CY1 complexes were visible in uninfected *N*. *benthamiana* samples. These results indicate that CY2-infected plants contain macromolecular assemblies resembling small VLPs that are associated with ORF5_CY2_ and CY2 RNA, supporting the hypothesis that ORF5_CY2_ has features more consistent with CPs than MPs.

### CY1 and a CY1-derived defective (D)-RNA bind phloem protein 2 (PP2) in vitro and in vivo and form large RNP complexes with cucumber sap that confers partial protection from RNase digestion

MPs are required for protecting the viral gRNA from degradation and for transport through plasmodesmata [11]. Since CY1 and ORF5_CY2_-deficient CY2 were able to systemically infect *N*. *benthamiana* (Fig 2 and Table 1) in the absence of an encoded MP or helper virus, the most likely explanation is that CY1 and CY2, similar to viroids, are using a host RNA MP. Since cucumber is an excellent model system for studying the movement of endogenous RNAs in the phloem due to the availability of large quantities of phloem sap [55,56], we agroinfiltrated cucumber with CY1 to assess its suitability as a host. RT-PCR of systemic leaves from infiltrated cucumber revealed the presence of CY1 (+)- and (-)-strand RNAs (S8 Fig), suggesting that cucumber would be a good system for identifying host proteins involved in CY1 systemic movement.

**Fig 7:**
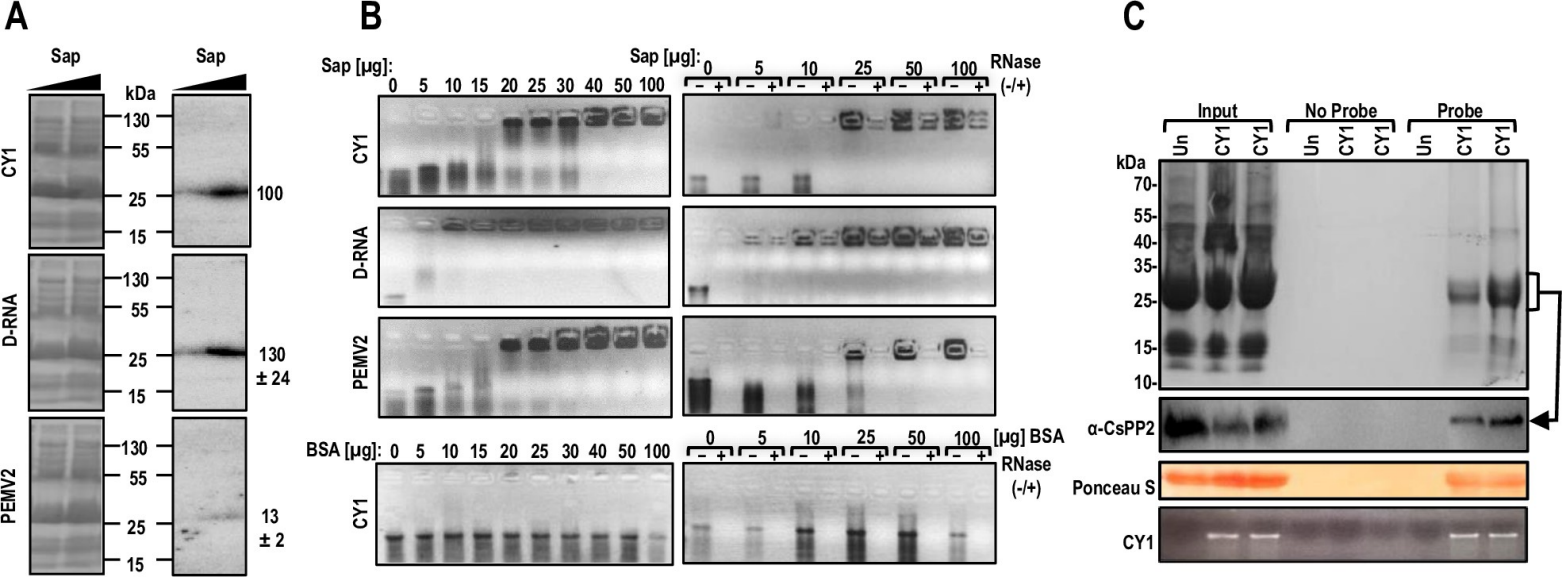
CY1 and a CY1-derived defective (D)-RNA binds to cucumber PP2 in vitro and in vivo, which confers partial protection against RNase. **(A)** Northwestern analyses of CY1-binding proteins in cucumber sap. Cucumber phloem exudates (25 and 100 μg) were subjected to electrophoresis on 15% SDS-PAGE gels that were stained with Coomassie blue (left), or transferred to nitrocellulose membranes and probed with uniformly labeled CY1, CY1 D-RNA, or PEMV2 transcripts (right). Numbers to the right denote relative binding in 3 independent experiments with standard deviations. **(B)** EMSAs. Left, transcripts were mixed with increasing amounts of cucumber phloem sap extracts and analyzed on 1.5% non-denaturing agarose gels containing ethidium bromide. Right, EMSAs of sap+transcripts that were treated (+) or not treated (-) with RNAse A at room temperature for 15 min. BSA in place of sap was used as a negative control. **(C)** Phloem sap exudates from uninfected (Un) and CY1-infected cucumber (2 samples) at 12 dpi were collected (Input) and used for RNA-pulldown assays using streptavidin beads without (No Probe) and with attached 5′-biotinylated CY1 probes (Probe). Pulldown samples were resolved by SDS-PAGE. Top panel: gel stained with Coomassie blue. Second panel, western blot using anti-cucumber PP2 antibody (α-CsPP2). Arrow denotes the 26 kDa band corresponding to PP2. Third panel: membrane from western blot stained with Ponceau S. Fourth panel, RNAs were extracted and subjected to RT-PCR using CY1-specific primers. dpi, days post-infiltration; EMSA, electrophoretic mobility shift assay; PEMV2, pea enation mosaic virus 2; PP2, phloem protein 2.

Northwestern assays were performed using cucumber phloem sap and uniformly labeled (i) CY1; (ii) a CY1 defective RNA (D-RNA; 921 nt) composed of positions 1–671 joined to positions 2442–2693 that is frequently found in CY1- and CY2-infected *N*. *benthamiana*; and (iii) umbravirus PEMV2 (Fig 7A). All viral RNAs bound to a single location in the gels, corresponding with the position of the major 26 kDa variant of PP2, the protein responsible for HSVd movement. Approximately 7.5-fold more CY1 and 10-fold more D-RNA bound at this location compared with umbravirus PEMV2, suggesting that the protein migrating at this position is binding more efficiently to CY1 RNAs.

To further investigate CY1/CY1 D-RNA specific interaction with a sap protein, electrophoretic mobility shift assays (EMSAs) were conducted using varying amounts of cucumber sap and sufficient RNA for detection using ethidium-bromide-stained gels. CY1 elicited a sharp transition to a high MW complex in the presence of 20 μg of sap that was unable to migrate into the 0.8% agarose gels (Fig 7B). Addition of 40 μg of sap shifted all detectable CY1 transcripts to the high MW complex. CY1 D-RNA transitioned nearly completely to the high MW complex with the addition of only 10 μg of sap. EMSAs of PEMV2 were similar to those of CY1, despite binding more weakly to the interacting protein(s) in the northwestern assays. To examine whether the high MW complex protects the associated RNA from treatment with RNase, EMSAs were repeated with and without added RNase A. A fraction of the CY1 complex and the majority of the CY1 D-RNA complex were protected from 15 min of RNase treatment at all sap concentrations (Fig 7B, right panel). In contrast, the complex containing PEMV2 showed no protection, even at the highest sap concentration. These results suggest that a protective RNP complex forms between sap proteins and CY1 RNAs.

To determine if CY1 is interacting with PP2 in vivo, RNA pulldown assays were performed. Sap from uninfected and CY1-infected cucumber at 12 dpi was collected and treated with a biotin-labeled probe complementary to CY1. Several proteins were extracted along with CY1, including a major protein of 24 to 26 kDa (Fig 7C, upper panel). Western blots demonstrated that this interacting protein cross-reacted with anti-cucumber PP2 antibody and RT-PCR confirmed that the pull-down complex contained CY1 RNA (Fig 7C, bottom panels). These results suggest that PP2, a 26 kDa protein with a vascular expression profile [57] that forms RNP complexes with HSVd in vitro [58], is also associating with CY1 in phloem sap during infection.

### Reducing *N*. *benthamiana* PP2 expression decreases CY1 accumulation in systemic leaves

**Fig 8:**
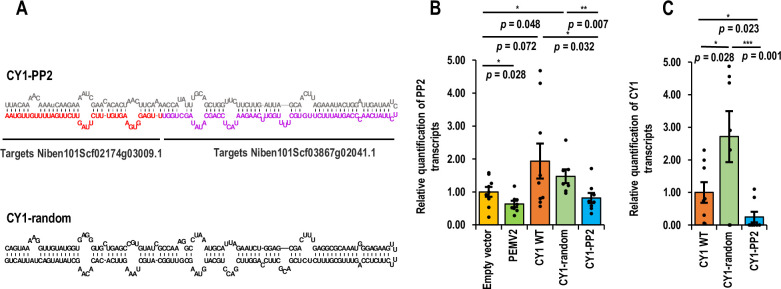
CY1 VIGS vector targeting mRNA of 2 PP2 family members decreases CY1 accumulation in systemic leaves. **(A)** Hairpin used to target the 2 PP2 mRNAs and similarly sized random hairpin. Hairpins replaced the natural hairpin at position 2220. Agrobacteria constructs harboring either PEMV2, CY1 WT, CY1-PP2, or CY1-random were agroinfiltrated into *N*. *benthamiana* leaves. Total RNA from systemic leaves was extracted for relative quantification of RNA at 28 dpi. **(B)** PP2-specific primers were used for quantification of PP2 relative to actin. **(C)** CY1-specific primers were used for quantification of CY1. Data were analyzed using GLMs as the data were neither normally distributed nor homoscedastic. The omnibus test (likelihood ratio chi-square test) gave *p* ≤ 0.012, rejecting the null hypothesis that treatments did not have any effect on accumulation. Significance was determined using differences at a *p*-value of * < 0.05, ** < 0.1, and *** < 0.001 as determined from an LSD test. Error bars represent standard error. Experiments were performed with *n* > 6 biological replicates per treatment. Data for relative quantification of PP2 and viral transcripts can be found in S1 Data Supporting information. dpi, days post-infiltration; GLM, generalized linear model; LSD, least significance difference; PEMV2, pea enation mosaic virus 2; PP2, phloem protein 2; VIGS, virus-induced gene silencing.

PP2, one of the most abundant proteins in cucumber phloem sap [59], contains a central domain with a jelly-roll topology similar to 30K MPs (S9 Fig). To determine if PP2 is important for systemic movement of CY1, CY1 was used as a VIGS vector to target PP2 mRNA for degradation by incorporating a PP2 mRNA-targeting hairpin (CY1-PP2) (Fig 8A). WT CY1, PEMV2, CY1-PP2, or CY1 with a non-targeting hairpin of the same size (CY1-random) were infiltrated into *N*. *benthamiana*, and levels of PP2 mRNA were assessed in systemic leaves using qRT-PCR. Interestingly, both WT CY1 and CY1-random enhanced the accumulation of PP2 mRNA relative to infection with PEMV2 (*p* = 0.01 and *p* < 0.001, respectively). In contrast, accumulation of PP2 transcripts decreased significantly when CY1-PP2 was infiltrated relative to WT CY1 (*p* < 0.05) and CY1-random (*p* = 0.007) (Fig 8B). Accumulation of CY1-PP2 also decreased significantly in systemic leaves relative to WT CY1 (*p* < 0.05) and CY1-random (*p* = 0.001) (Fig 8C). Correspondingly, CY1-PP2-infected plants displayed reduced symptoms with larger, greener leaves, and less stunting (S10 Fig). These results suggest that PP2 is important for systemic movement of CY1 and very likely also CY2.

## Discussion

It is widely accepted that viruses must encode at least 1 MP to establish a successful systemic infection of plants. This general assumption is built upon extensive studies of numerous plant viruses that showed no systemic infection when the encoded MP was significantly altered or absent [1,11–14]. For this reason, it was assumed that at least one of the non-replication proteins encoded by Class 2 or Class 3 ULVs was an MP [39]. Similarly, it was expected that ULVs would require a helper virus to support infection as is observed for umbraviruses, which lack a CP and silencing suppressor. Curiously, several reports using next-generation sequencing did not identify a helper virus in plants infected with Group 2 ULVs, despite specific attempts to do so [40,44,60]. We have found that agroinfiltration of CY1 in *N*. *benthamiana*, citrus, and cucumber results in a systemic infection that occurs in the absence of any additional viruses (Figs 2, S2, and S8 and Table 1), as does infiltration of CY2 onto *N*. *benthamiana*. These results thus support the published observations that CY1 and CY2 are accumulating independent of a helper virus in natural hosts.

All Class 2 ULVs with the exception of CY1 encode an additional protein (ORF5) with a single jelly-roll domain, which is a conserved structural feature of both 30K MPs and related CPs (Fig 5) [8]. Lack of ORF5, either naturally in CY1 or engineered in CY2, delayed symptoms in plants relative to WT CY2 (Table 1). CY1 infection of transgenic *N*. *benthamiana* expressing ORF5_CY2_ or ORF5_OULV_ was more rapid than infection of WT plants, with symptoms appearing in most plants by 2-wpi similar to CY2, rather than 3-wpi, as found for CY1 infection of WT plants (Table 2). ORF5 protein was originally proposed to be an MP based on similar gene organizations of ULVs and umbraviruses, and the lack of reports of any plant viruses without an encoded MP [39]. Based on our analyses, we propose that ORF5 has features more consistent with CPs than canonical MPs, and that CY1 and CY2 do not require either an encoded MP or helper virus for systemic infection. This proposal is based on our examination of ORF5_CY2_, finding that: (i) ORF5_CY2_ and other ORF5 proteins do not contain the invariant aspartic acid residue present in 30K MPs (S7A Fig); (ii) ORF5_CY2_ and other ORF5 proteins have an asymmetric distribution of positively charged residues, similar to CPs (S7B Fig); (iii) ORF5_CY2_ and other ORF5 proteins have a closer evolutionary relationship with LPS CPs than with MPs (Fig 5D); (iv) ORF5_CY2_ is connected with the presence of macromolecular structures in infected plants that resemble VLPs, which are associated with CY2 RNA and ORF5_CY2_ protein (Fig 6); and (v) ORF5_CY2_ is not required for systemic infection of CY1 or CY2, but rather supports virus movement by shortening the timing to initial symptoms (Tables 1 and 2). Several CPs are also known to support virus movement by interacting with the RNP complex [13,27,61–63] or by suppression of host defenses [31,64]. Thus Class 2 ULVs may resemble the original plant viruses that would have lacked encoded MPs and which were believed to no longer be present in the contemporary virome [8]. Finding VLPs in CY2-infected plants may also explain the lack of helper viruses for transmission from plant to plant if these are infectious particles. This latter hypothesis awaits confirmation by future transmission studies.

Interestingly, EM images revealed different forms of VLPs in CY2-infected plants, with the major form having a diameter of about 14 nm and 2 less abundant forms of 24 and 35 nm. Importantly, none of these particles were seen in samples from uninfected or CY1-infected *N*. *benthamiana*. One possibility is that the larger forms represent swollen particles, which is characteristic of sobemovirus virion particles, where virion stability depends on pH and the availability of divalent cations [65–68]. A common ancestor giving rise to ORF5 and sobemovirus SeMV CP has very high support (91%, node B) with lower support for related luteovirus and polerovirus CPs (Fig 5). All of these CPs share with ORF5_CY2_ the feature of having an N-terminal NoLS [52,69,70].

Systemic infection of plants by CY1 and CY2 ORF5 mutants, which only express replication-associated proteins, strongly suggests that both CY1 and CY2 make use of a host RNA MP. CY1 and the commonly derived D-RNA interacted with a protein in northwestern gels, which co-migrated with the most abundant sap protein, the 26 kDa isoform of PP2 (Fig 7A). PP2, expressed only in phloem CCs [71], is described as an ancient, highly conserved protein present in all higher plants. PP2 forms filamentous structures when crosslinked to phloem protein 1, which are important for impeding the entry of bacterial and fungal pathogens and restricting their movement in phloem sieve tubes [72,73]. Similar to 30K viral MPs, the 26 kDa PP2 isoform contains a single jelly-roll domain [74], increases the SEL of PD [75], and transits through sieve tubes complexed with host mRNAs [76]. In addition, this PP2 isoform has been implicated in the systemic movement of circular RNA viroids [58,59]. Interaction between cucumber PP2 and CY1 in vivo was supported by pulldown assays performed with phloem sap exudates from infected cucumber plants (Fig 7C). Furthermore, silencing this PP2 isoform significantly inhibited CY1 accumulation in systemic leaves of *N*. *benthamiana* (Fig 8). WT CY1 and CY1 carrying a random hairpin (CY1-random) increased PP2 transcripts compared with levels in plants infiltrated with Agrobacteria carrying an empty Ti plasmid (Fig 8B), which would increase the supply of PP2 available for movement. Interestingly, this increase was not observed in PEMV2-infected plants, which uses encoded MPs. Sap partially or fully protected CY1 and D-RNA against RNase A treatment, respectively, but not PEMV2, suggesting that PP2 and possibly other sap proteins are interacting in a specific manner with and fully coating CY1 and D-RNA (Fig 7B). CY1-infected plants contained numerous irregularly shaped complexes when visualized by EM (Fig 5D); however, we were unable to detect CY1 RNA associating with this material (Fig 5C). Currently, the mechanism by which PP2 protects CY1 and D-RNA remains unresolved.

In conclusion, the present work describes the first plant virus that despite not encoding an MP is capable of independent systemic infection with the likely assistance of a host MP. It would be of interest to ascertain if MP-encoding viruses might also make use of host MPs for long-distance movement in plants.

## Materials and methods

### Plasmid constructs

CY2 (also known as CYVaV-δ; accession number MT893741) [77] was synthesized by Twist Bioscience (California, United States of America) in vector pTwist downstream of a T7 promoter and preceded by a HindIII restriction site. The GenBank CY2 sequence has an incorrect 5′ end, and the 63 nt of 3′ end sequence located upstream of the correct start site at position 64 were removed. Moreover, the GenBank sequence also stops short of full-length. Therefore, we used sequence from CY1 and another hemp CY2 (MT893740.1) to complete the 3′ end (16 nt). For agroinfiltration, CY2 was cloned into binary vector pXT1 (Gene Bank accession number JN029690) developed from pCB301 [78], generating the construct pCB301-CY2. Earlier the same binary vector, pCB301, was used to clone CY1 (pCB301-CY1) [39]. Ligation independent cloning (LIC) was used to introduce mutations into the CY2 genome, which were confirmed by DNA sequencing. Plasmid pCB301-TMV-GFP was generated by LIC from TTU2T [79] and pXT1. The truncated TMV MP construct was created using PCR as described previously [80]. A variant of TMV-GFP with a deleted CP was similarly generated. LIC was also used to replace the TMV MP or CP with CY2_ORF5_. For plant transformation, ORF5_CY2_ or ORF5_OULV_ sequences were inserted between the XmaI and SacI of pBI121 (Gene Bank accession number AF485783) [81]. RFP-NoLS was amplified from pSAT6-mCherry-VirD2NoLS [82], which was obtained from the Arabidopsis Biological Resource center (stock no. CD3-1106), and was inserted into pBI121 between the XmaI and SacI sites. To determine localization of ORF5 protein in plant cells, ORF5_CY2_-GFP was constructed by inserting ORF5_CY2_ into pBI121 between the XbaI and XmaI sites, followed by insertion of mGFP5-ER between the XmaI and SacI sites. For construction of CY1-PP2 and CY1-random, HiFi DNA assembly kit (NEB) was used to introduce the PP2-targeting hairpin or a similar “random” hairpin of the same length (198 nt) at position 2220 of CY1 in pCB301-CY1 plasmid, which replaces the natural hairpin at that location. See Fig 8A for hairpin sequences.

### Syringe and vacuum infiltration of *Agrobacteria tumefaciens*

Electrocompetent *Agrobacterium tumefaciens* strain GV3101 was transformed with binary vectors using electro cell manipulator ECM 395 (BTX) and then plated on LB with antibiotics (20 mg/l rifampicin and 50 mg/l kanamycin) for 72 h at 28°C. A single colony was transferred to 3 ml of LB supplemented with antibiotics and incubated overnight with shaking at 28°C. The seed culture was transferred to 10 ml of fresh LB with antibiotics until an OD_600_ of 0.8 was reached. The culture was centrifuged at 4,000 g for 15 min, and precipitated cells were resuspended in infiltration MMA buffer (10 mM MES (pH 5.6), 10 mM MgCl_2_, 100 μm acetosyringone) with the final concentration adjusted to an OD_600_ of 0.4. The infiltration culture was incubated at room temperature for 2 h prior to infiltration using a 1 ml needleless syringe applied to the abaxial surface of leaves 3 and 4 at the 6 to 8 true leaf stage *N*. *benthamiana* plants, or both cotyledons for cucumber. All infiltrations also included pCB301 expressing the p14 silencing suppressor of pothos latent virus. To determine the localization of ORF5_CY2_ or ORF5_OULV_ in plant cells, agrobacteria containing either ORF5_CY2_-GFP or ORF5_OULV_ -GFP (OD_600_ of 1.2) was mixed with RFP-NoLS at a ratio of 1:1, followed by syringe infiltration. Agrobacterium with CY1, CY2, CY2-derived mutants, and p14 were cultured in large volume overnight and the OD adjusted with MMA. Plants were immersed upside down when applying the vacuum.

### Transfection of protoplasts and RNA extraction

Callus cultures of *A*. *thaliana* (ecotype Col-0) were used to prepare protoplasts as described previously [83]. Protoplasts (5 × 10^6^) were transfected with 20 μg of CY1, CY1_GDD_, CY2, CY2sgm1, CY2sgm2, and CY2T5 transcripts synthesized by T7 RNA polymerase using linearized plasmids. Polyethylene glycol 1450 was used to mediate transfection. Protoplasts were collected at 24- and 48-hpi and total RNA was extracted with buffer (50 mM Tris-HCl (pH 7.5), 5 mM EDTA (pH 8.0), 100 mM NaCl, and 1% SDS), followed by phenol-chloroform treatment and isopropanol precipitation [39]. Transfections were repeated 3 times independently.

### RNA detection by RT-PCR and quantification by RT-qPCR

For *N*. *benthamiana* and cucumber, plant tissue (100 mg) was collected from individual plants and RNA extraction was performed using Trizol (Invitrogen) following the manufacturer’s protocol. Total RNA (200 ng) was used to synthesize cDNA using M-MuLV reverse transcriptase (New England Biolabs) and random primers. For *N*. *benthamiana*, PCR (25 cycles) was conducted using CY1-specific primers 5′-CATGGGCTCGCTGAGTAAAATAGAA and 5′-GCAGACTGTCCAAGACCTAAAGGAC, amplifying positions 2056 to 2401. For cucumber, PCR (27 cycles) was performed using CY1-specific primers 5′-CGAGCATCAACTAGCTTCTCAAGAG-3′ and 5′-GATGACAATTAAGCTACCAG, amplifying positions 63 to 2469. PEMV2 primers were 5′-GCTCCGTACTGAAGCGGTT and 5′-TGCGAAGTTGCCTTCGAA, amplifying positions 2950 to 3251. Viral RNA and host transcripts were quantified relative to actin transcripts using reverse transcription quantitative real-time PCR (RT-qPCR). All primers used for quantification are available upon request. RT-qPCR was performed using a 15 μl mixture containing SYBR Green PCR Master Mix (Applied Biosystems, Foster City, California, USA). Thermocycling conditions were: 2 min polymerase activation at 50°C followed by initial denaturation for 2 min at 95°C and 45 cycles at 95°C for 15 s, 55°C for 1 min. Each sample was quantified in triplicates and no-template control was included. Analysis of RT-qPCR fluorescence data was performed and expressed in fold change relative to actin using the ΔΔCT method.

### RNA gel blots

Two micrograms of total RNA extracted from *N*. *benthamiana* plants or *Arabidopsis* protoplasts were denatured at 65°C for 2 min, subjected to electrophoresis on 1% denaturing agarose gels, and transferred to a nitrocellulose membrane (Amersham Hybond ECL- GE Healthcare). The membrane was subjected to UV crosslinking, and then probed using a mixture of 3 [γ-^32^P] ATP-labeled oligonucleotides complementary to CY1 positions 2202–2239, 2464–2502, and 2655–2692. Blots were exposed to a phosphorimaging screen and imaged on a Typhoon scanner. Band intensities were quantified using GelQuantNet software.

### Fluorescence in situ hybridization and confocal microscopy

FISH was performed as previously described with modifications [84]. Stem and root tissues of *N*. *benthamiana* plants were manually excised into approximately 150 μm diameter sections using a sterile razor blade and then fixed overnight at 25°C in 4% paraformaldehyde in PBS buffer (145 mM NaCl, 2.7 mM KCl, 8.1 mM Na_2_HPO_4_, 1.5 mM KH_2_PO_4_ (pH 7.4)). Fixed samples were washed in PBS buffer for 10 min, followed by dehydration in an ethanol series (15%, 50% 70%, 96%, 100%) for 30 min each with shaking at 50 rpm (25°C). Dehydrated samples were then washed with ice-cold PBS buffer for 3 min and suspended in hybridization buffer containing probe (0.9 M NaCl, 20 mM Tris-HCl (pH 7.5), 0.01% SDS, 30% formamide, 4 ng/μl Cy3-labeled oligonucleotides targeting CY1 position 1301–1351) for 24 h at 25°C. Samples were then washed with pre-warmed washing buffer (102 mM NaCl, 20 mM Tris-HCl (pH 7.5), 5 mM EDTA (pH 8.0)) at 43°C for 15 min in the dark. Samples were rinsed with ice-cold sterilized nuclease-free water and transferred to a microscope glass-slide. After removal of excess water, samples were mounted with ProLong Gold Antifade mountant with DAPI (Thermo Fisher). FISH slides were observed and imaged using a Zeiss LSM710 confocal laser-scanning microscope with Zen software.

### Plant transformation

Fully expanded *N*. *benthamiana* leaves from 8-true leaf stage plants were sterilized using 0.5% sodium dichloroisocyanurate for 30 min and then cut into small pieces. Leaf disks were incubated with Agrobacteria for expression of ORF5_CY2_ or ORF5_OULV_, which were resuspended in Murashige and Skoog medium (MS) for 30 min. Leaf disks were then transferred onto a co-culture medium (MS media containing 1.2 mg/L 6-benzylaminopurine [6-BA] and 0.1 mg/L a-naphthalene acetic acid) and petri dishes were incubated in the dark for 2 days at room temperature. Transformants were selected on selective medium (MS media containing 1.2 mg/L 6-BA, 0.1 mg/L NAA, 200 mg/L kanamycin, and 250 mg/L cefotaxime). Shoots induced from callus were excised and transferred to rooting media (MS media containing 0.1 mg/L NAA, 200 mg/L kanamycin, and 250 mg/L cefotaxime). Well-developed shoots along with roots were transferred to the soil under 16 h light at room temperature until seed set. Seeds were screened on MS medium containing 200 mg/L kanamycin and 250 mg/L cefotaxime.

### Cucumber growth conditions and phloem exudate sampling

Cucumber (*Cucumis sativus* cv. Spacemaster) was grown in Promix growing medium-LP15 under long-day conditions (16 h light). Fertilizer was added weekly by drenching the soil with a solution of all-purpose Miracle-Gro plant food (20-20-20 N, P and K, plus minor elements). For phloem exudate collection, cucumber plants were cut between the cotyledons and the first 2 true leaves to collect the exudates, which were then diluted 1:5 in phloem extraction buffer (PEB) (80 mM Tris-HCl (pH 8.0), 1 mM EDTA). All experiments with exudates were conducted within 2 h of sap collection.

### Northwestern assays

Phloem exudates from cucumber were mixed with 2X Laemmli sample buffer, heated for 5 min at 95°C, subjected to electrophoresis on denaturing 15% SDS-PAGE gels, and then transferred to nitrocellulose membranes (Bio-Rad). Membranes were stained with Ponceau S, photographed, rinsed 3 times with water and then submerged in northwestern buffer (10 mM Tris-HCl (pH 7.5), 1 mM EDTA, 100 mM NaCl, 0.02% w/v Ficoll 400, 0.02% w/v PVP-40, 1% BSA) for 2 h at 25°C with gentle shaking to renature the proteins. Membranes were then hybridized for 3 h in northwestern buffer supplemented with 5 pmol of ^32^P-labeled probe and 50 μg/ml of yeast tRNA. Following hybridization, membranes were washed 5 times with NW buffer for 10 min each and imaged using an Amersham Typhoon Gel Blot Imaging System (GE Healthcare). Comparative binding levels were determined after accounting for the length and incorporation of radiolabeled nucleotides into the probes.

### Electrophoretic mobility shift assays

pUC19 constructs containing full-length CY1, CY1 D-RNA, or PEMV2 were digested downstream from the inserts and used as templates for RNA synthesis using T7 RNA polymerase. Phloem sap extracts were diluted 1:5 in ice-cold PEB and the protein concentration was determined by absorbance in a Denovix DS-11 spectrophotometer. EMSA reactions (10 μl total volume) were prepared using 5 μl of 2X binding buffer (50 mM sodium-phosphate, 50 mM NaCl), 4 μl of phloem sap extracts (from 5 to 100 μg), and 0.35 pmol of in vitro transcribed RNA. Samples were incubated on ice for 20 min, followed by 15 min at 25°C either with or without 0.02 μg of RNase A. Samples were immediately loaded onto 1.5% non-denaturing agarose gels containing ethidium bromide for visualization.

### CY1 pulldown assays

The 5′-biotin labeled CY1 probes were biotin-GGGCGCUGUUAACUC (positions 2678–2692) and biotin-CGAAGGGUGAGGCCC (positions 2488–2502). Probes were bound to 50 μl of prewashed streptavidin magnetic beads at 4°C for 1 h according to the manufacturer’s instructions (Dynabeads MyOne Streptavidin C1-Thermofisher) using a modified version of Interaction Buffer (20 mM Tris-HCl (pH 7.5), 300 mM KCl, 0.2 mM EDTA, 0.5 mM DTT, 0.5 mM phenylmethylsulfonyl fluoride) as previously described [85]. During this step, phloem sap extracts were collected in triplicate: 0.5 ml of the phloem sap extract was diluted into 1 ml of RNA Pulldown Buffer (20 mM Tris-HCl (pH 7.5), 150 mM NaCl, 1 mM MgCL_2_, 1 mM CaCL_2_, 0.1% SDS, 1% sodium deoxycholate, 1% Triton X-100, 5 mM PMSF, 5 mM DTT, Protease Inhibitor 5 mg/ml [Pierce], and Murine RNase Inhibitor [50 U/ml; NEB]) and kept on ice. Streptavidin magnetic beads were added to samples with and without the biotinylated probes, mixed gently by pipetting, and incubated at 4°C for 4 h with gentle rotation (10 rpm). Incubated mixtures were placed in a magnetic stand for 1 min to isolate the magnetic beads, followed by 2 washes of the beads with 500 μl of washing buffer (20 mM Tris-HCl (pH 7.5), 100 mM KCl, 2 mM EDTA, 0.5 mM DTT, and 0.5 mM PMSF) and then 2 washes with cold interacting buffer, isolating the beads with the magnetic stand in-between washes. Of the triplicate samples, one was subjected to Trizol-RNA extraction, cDNA synthesis, and RT-PCR for detection of CY1. The remaining samples were treated with 1 μg of RNase A, the beads isolated and added to 20 μl of Laemmli sample buffer (126 mM Tris-HCl (pH 6.8), 20% glycerol, 4% SDS, and 0.02% bromophenol blue) and subjected to SDS-PAGE for visualization and immunodetection with cucumber PP2 antibody.

### Western blots

Phloem exudates from cucumber (non-infected or infected), or pulldown samples were collected, fractionated on 14% SDS-PAGE gels, transferred to nitrocellulose membrane, blocked with TBS buffer (500 mM NaCl, 20 mM Tris (pH 7.5), 0.1% Triton X-100) supplemented with 1% bovine serum albumin and incubated overnight with polyclonal antiserum to cucumber PP2 (1:3,000) [9]. Membranes were washed 3 times for 15 min each with TBS buffer with 0.1% Tween-20, followed by a 2 h incubation with the secondary antibody (anti-rabbit immunoglobulin linked to horseradish peroxidase (HRP) [1:5,000]; Sigma-Aldrich) and visualized by chemiluminescence using the Perkin-Elmer Life Sciences reagent according to the manufacturer instructions.

### Virus-induced gene silencing of PP2 mRNA

A 198 nt hairpin was designed to silence the expression of 2 *N*. *benthamiana* PP2 transcripts (Niben101Scf03867g02041.1; Niben101Scf02174g03009.1), which are orthologues of the cucumber PP2 previously found to have a role in viroid movement [9,86,87]. After vacuum infiltration of WT CY1, PEMV2, CY1-PP2, and CY1-random, total RNA was extracted from systemic leaves at 28 dpi for quantification of viral RNA by RT-qPCR.

### Protein structure prediction and analysis

ORF5_CY2_, PP2, MP_OuMV_, and CP_BYDV_ were modeled using AlphaFold2 [88]. ORF5_CY2_ was superimposed on MP_OuMV_ using TM-align [89]. Structural similarity between ORF5_CY2_ and MP_OuMV_ was further evaluated based on TM scores, using 0.5 as a cut-off. Superimposed images were exported from TM-align and loaded into Chimera X [90] for further processing. Structural similarity between ORF5_CY2_ and CP_BYDV_ was discovered using the DALI server [91] based on the highest Z score.

### VLP isolation and electronic microscopy

VLPs were isolated as described previously [54]. Briefly, 5 g of leaves were ground in liquid nitrogen and added to 20 ml of 0.1 M sodium phosphate (pH 7.2) supplemented with protease inhibitor cocktail tablets (Thermo Fisher Scientific, A32955). The homogenate was filtered through Miracloth followed by centrifugation to remove plant debris. Supernatant (10 ml) was added to an ultracentrifuge tube (Beckman, 331374) on top of 1 ml of 25% sucrose and 0.5 ml of 70% sucrose in 0.1 M sodium phosphate (pH 7.2). Ultracentrifugation was performed at 40,000 rpm for 2.5 h at 4°C. The 70% sucrose fraction was collected and treated with protein concentrator (Thermo Fisher Scientific, Cat 88513) to exchange the PBS buffer. The solution (5 μl) was applied onto a formvar-coated grid for 10 min and wicked to a thin layer of liquid. Five microliters of 2% glutaraldehyde was applied for 30 s, and liquid was removed with bibulous paper. The sample grid was washed with 5 μl of double distilled water by wicking off the liquid. Five microliters of 2% aqueous uranyl acetate was applied for 20 s and wicked to dryness. Grids were examined and imaged in a Hitachi SU-3500 scanning electron microscope.

### Phylogenetic analyses

Maximum-likelihood phylogenetic tree was used to evaluate the relationships of ULVs to all known umbraviruses based on RdRp nucleotide sequences. Alignments were generated and trimmed in MEGA X [92]. General time reversible model with gamma distribution rates and invariable frequency (GTR+G+I) was determined to be the best-fit substitution model in MEGA X and was implemented to construct the tree. The following ULV sequences were used: PMeV2 (KT921785), PpVQ (MT113180), BabVQ (MT113182) [42,93,94], OULV (MH579715, MT909563, MT909562) [44], FULV (MW480492, MW480492) [60], CY1 (JX101610) [40], CY2 (MT893741) [77], WULV (OK573479) [95], GULV(OP886321) [96], PIULV (OL330774) [97], SbaVa (MK211274) [98], TULV (OK018180) [99], MULV (OM937759) [41], JgULV (OM937760) [41], SULV (MN868593) [100], EMaV (MF415880), AgULV (OP660856), GPpTV1 (OM514399), ArULV (OQ102001) [101]. To analyze the phylogenetic relationship of ORF5 proteins with selected CPs and MPs, protein sequences were aligned using PROMALS3D [53] and trimmed in MEGA X. The LG model with gamma distribution rates and invariant frequency was determined to be the best-fit substitution model in MEGA X and was implemented to construct the tree.

## Supporting information

S1 Data Raw data for Tables 1 and 2 and Fig 8B and 8C. (XLSX)

S1 Raw Images Raw images of all gels can be found at https://figshare.com/search?q=10.6084%2Fm9.figshare.25407199. (PDF)

S1 Fig Umbra-like viruses contain 3′ RNA structures similar to umbraviruses. **(A)** Genome organization of umbraviruses, Group 1 umbra-like viruses (ULVs), and Group 2/Class 2 ULV CY1. **(B)** Secondary structure and tertiary interactions either known or predicted to occur at the 3′ terminus of ULVs and umbraviruses. Tertiary interactions are color coded. Conserved residues are in red. PEMV2, umbravirus pea enation mosaic virus; ArULV, arborvitae umbra-like virus (OQ102001); AgULV, Ageratum virus 1 (OP660856); CY1, Citrus yellow vein associated virus 1 (JX101610).
(PDF)

S2 Fig CY1 systemically infected Mexican lime in the absence of a helper virus. **(A)** Two approaches that were used to infect Mexican lime with CY1. Upper panel, two-week-old *N*. *benthamiana* were infiltrated with Agrobacterium containing a CY1-expressing Ti plasmid [step 1]. Fourteen days after infiltration, CY1-positive plants were colonized by dodder vines [step 2]. After detection of CY1 in dodder vines by RT-PCR, dodder tips were guided to colonize 2-month-old Mexican lime [step 3]. Lower panel, leaves and stems of 2-month-old Mexican lime were abraded using a Derma microneedle roller (Amazon:B0CH7SHD5T), and then vacuum-infiltrated with Agrobacterium containing a CY1-expressing Ti plasmid. **(B)** Right, northern blot used to detect CY1 in Mexican lime 15 months after dodder transfer or 12 months after vacuum infiltration. Left, infected plants at these times, and subsequently, did not display discernable systems. N.b., *Nicotiana benthamiana*; M.L., Mexican lime.
(PDF)

S3 Fig CY2 and CY2sgm1 accumulated in phloem tissues of stem, petiole, and roots. FISH analysis of CY1, CY2, and CY2sgm1 at 60 dpi. Cy3-labeled oligonucleotide probe targeted CY1 and CY2 positions 1101–1131. DAPI-stained DNA and xylem tissue fluoresce blue. Bar = 50 μm. No viral RNA signals were detected in non-phloem tissue.
(PDF)

S4 Fig *N*. *benthamiana* transgenic plants expressing ORF5 proteins. Western blot detection of ORF5_CY2_ and ORF5_OULV_. CY2 is the closest related ORF5-expressing ULV to CY1 and OULV is the least related of the dicot-infecting ULVs. Total proteins were isolated from heterozygous T1 plants and subjected to 10% denaturing PAGE. Primary antibody was developed in rabbits against peptide SLVRETYIPSSTTTGKE. ORF5 proteins are migrating as dimers under the conditions used.
(PDF)

S5 Fig ORF5_CY2_ does not complement MP-defective TMV-GFP or CP-defective TMV-GFP. **(A)** Schematic diagram of vectors used to infiltrate *N*. *benthamiana*. **(B)** Representative plants at 14 dpi. Only plants infiltrated with TMV-GFP show GFP expression in systemic leaves.
(PDF)

S6 Fig ORF5 protein contains a NoLS. **A.** NoLS (underlined) at the N-terminal region of ORF5 proteins in Class 2 ULVs. Asterisks denote conserved amino acids. Sequence was predicted using NoLStradamus; http://www.moseslab.csb.utoronto.ca/NoLStradamus/. **(B)** NoLS (underlined) at the N-terminal region of CPs of viruses from polerovirus (PLRV), luteovirus (BYDV), and sobemovirus (SeMV) genera.
(PDF)

S7 Fig ORF5 proteins have features inconsistent with canonical 30K MPs. **(A)** Alignment of ORF5 proteins with selected 30K MPs. Critical 30K MP aspartic acid residue (D) is in red. Bottom row denotes the consensus secondary structure predicted in PROMALS3D where e represents a β-strand. **(B)** Charge distribution for ORF5_CY2_, CP_BYDV_, and MP_OuLV_ by amino acid residue position (window size = 5) analyzed in EMBOSS CHARGE version 6.6.0. Red box denotes the jelly-roll domain. Note that charged residues are concentrated in the N-terminal region of ORF5_CY2_ and CP_BYDV_ but not MP_OuLV_.
(PDF)

S8 Fig CY1 systemically infects cucumber. **(A)** Schematic representation of a cucumber plant (*Cucumis sativus* cv. Spacemaster) agroinfiltrated with CY1 on cotyledons (solid green). Systemic leaves (numbered 1 to 4) were used to test for the presence of CY1. **(B)** Representative RT-PCR detection of CY1 in systemic leaves of 4 cucumber plants (Cs-1 to Cs-4) at 14 dpi. Negative control was the reaction in the absence of template and positive control was plasmid DNA containing full-length CY1. **(C)** Strand-specific detection of (+)- and (-)-strand CY1 in systemic leaves of 8 cucumber plants infiltrated with CY1 at 21 dpi. (-)-strand and (+)-strand lanes used strand-specific primers and plasmid DNA containing full-length CY1.
(PDF)

S9 Fig Structural similarity between the central domains of the ourmia melon virus MP (MP_OuMV_) and cucumber PP2. (PDF)

S10 Fig CY1-mediated silencing of *PP2* mRNA results in less severe symptoms on *N*. *benthamiana*. Photos of 4 plants each infected with WT CY1, CY1-random, and CY1-PP2 were taken at 50 dpi.
(PDF)

## Abbreviations

BYDV: barley yellow dwarf virus
CCS: carmovirus consensus sequence
CP: capsid protein
dpi: days post-infiltration
EM: electron microscopy
EMSA: electrophoretic mobility shift assay
FISH: fluorescence in situ hybridization
hpi: hours post-transfection
HRP: horseradish peroxidase
HSVd: hop stunt viroid
LIC: ligation independent cloning
LPS: luteovirus/polerovirus/sobemovirus
MP: movement protein
ORF: open reading frame
OULV: opuntia umbra-like virus
PD: plasmodesmata
PEB: phloem extraction buffer
PEMV2: pea enation mosaic virus 2
PP2: phloem protein 2
PPC: phloem parenchyma cell
RdRp: RNA-dependent RNA polymerase
RNP: ribonucleoprotein
SEL: size exclusion limit
ULV: umbravirus-like virus
VIGS: virus-induced gene silencing
VLP: virus-like particle
wpi: weeks post-infiltration
WT: wild type
