# Supplementary material for: Umbravirus-like RNA viruses are capable of independent systemic plant infection in the absence of encoded movement proteins
Source: PLoS Biol. 2024 Apr 25;22(4):e3002600. doi: 10.1371/journal.pbio.3002600 (PMC11081511; doi:10.1371/journal.pbio.3002600)

2C_raw_image

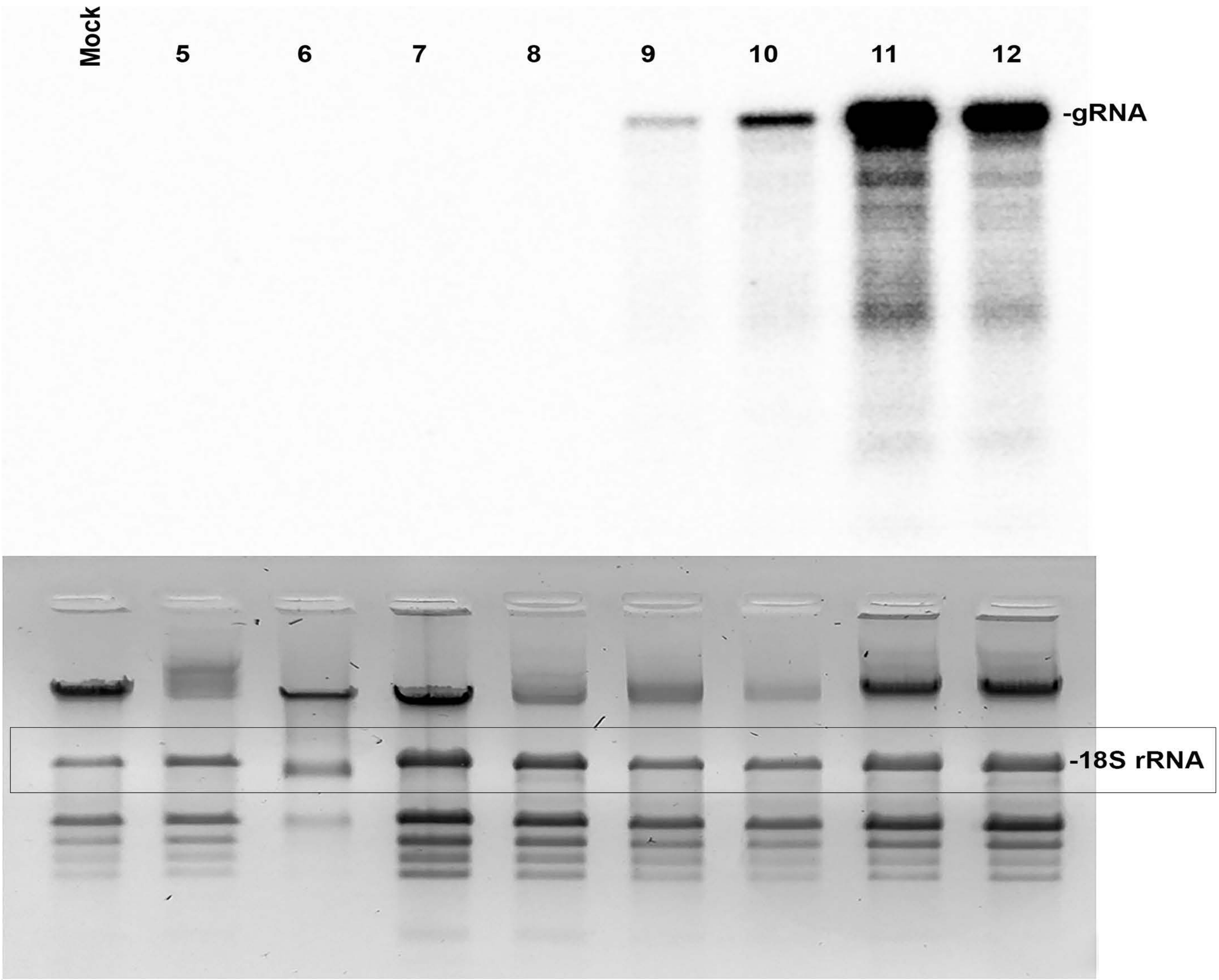

2D_raw_image

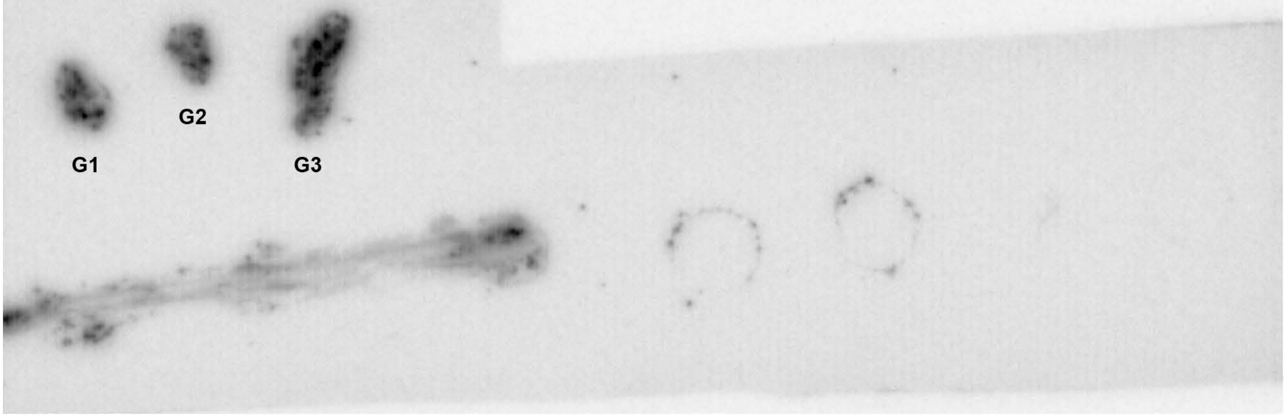

4B_raw_images

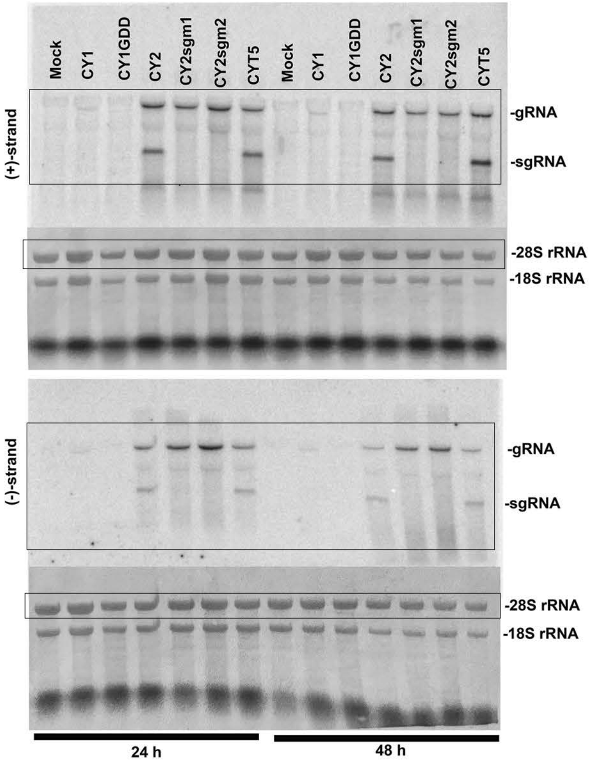

4c_raw_images

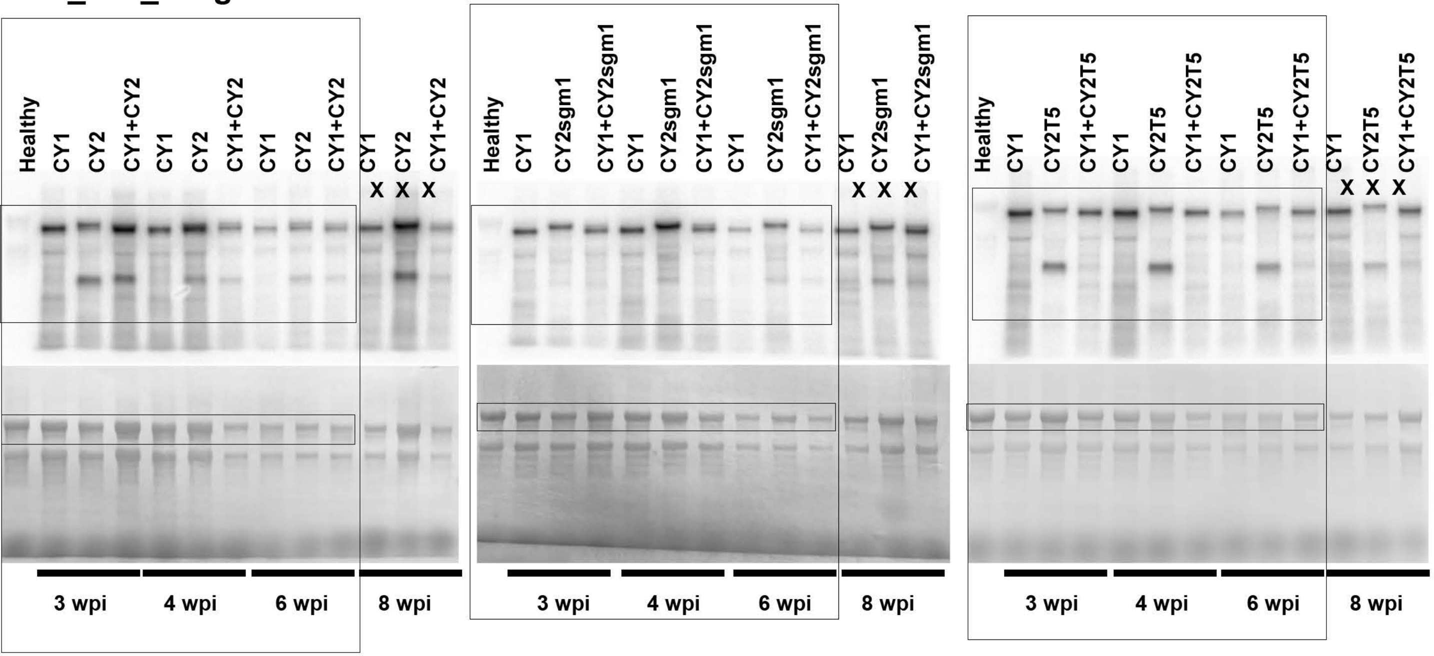

6A_raw_image

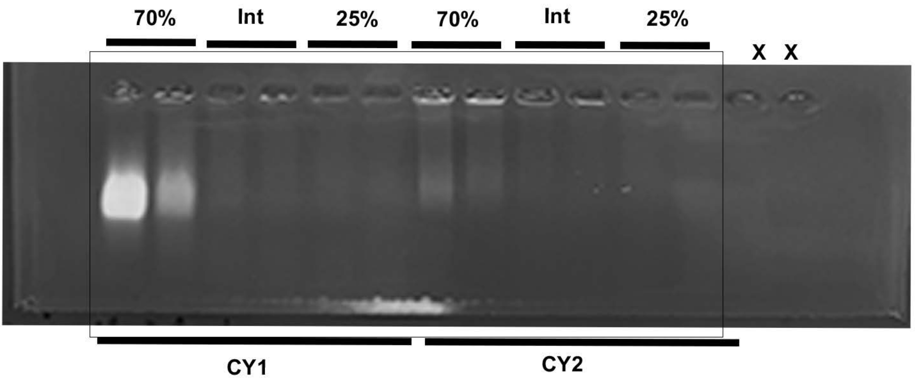

6B_raw_image

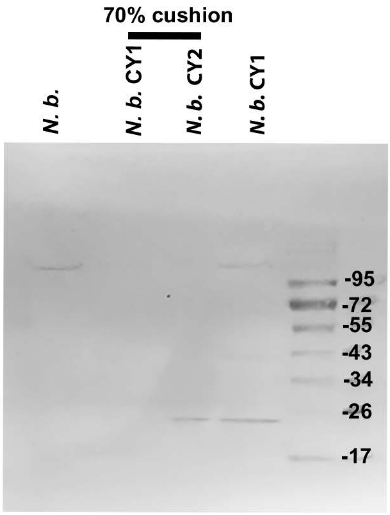

6C_raw_image

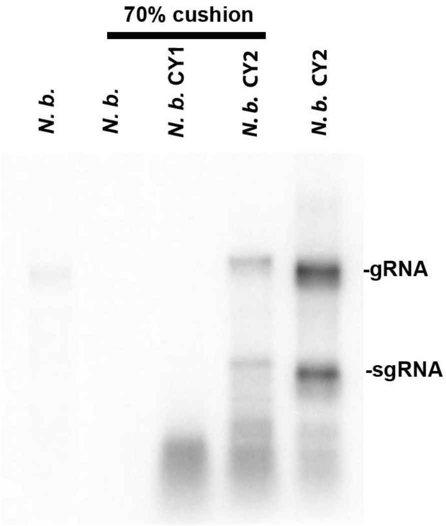

7A_raw_images

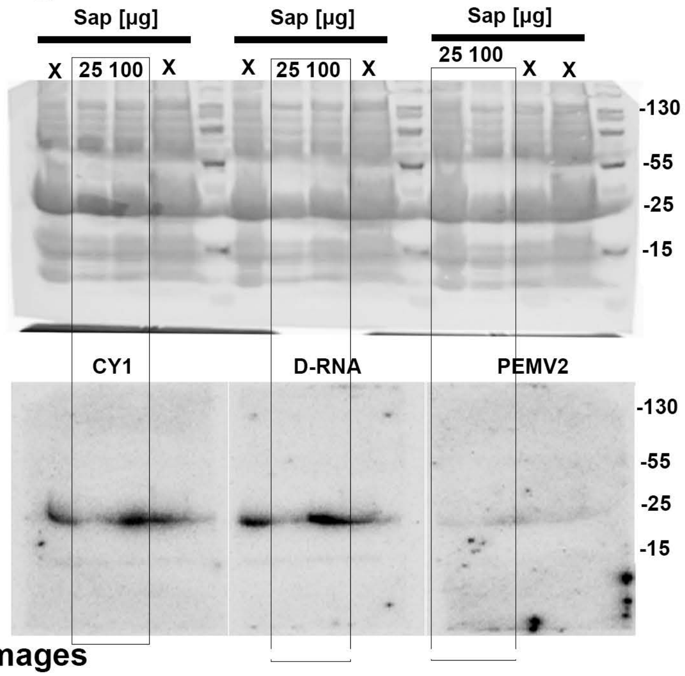

7B_raw_images

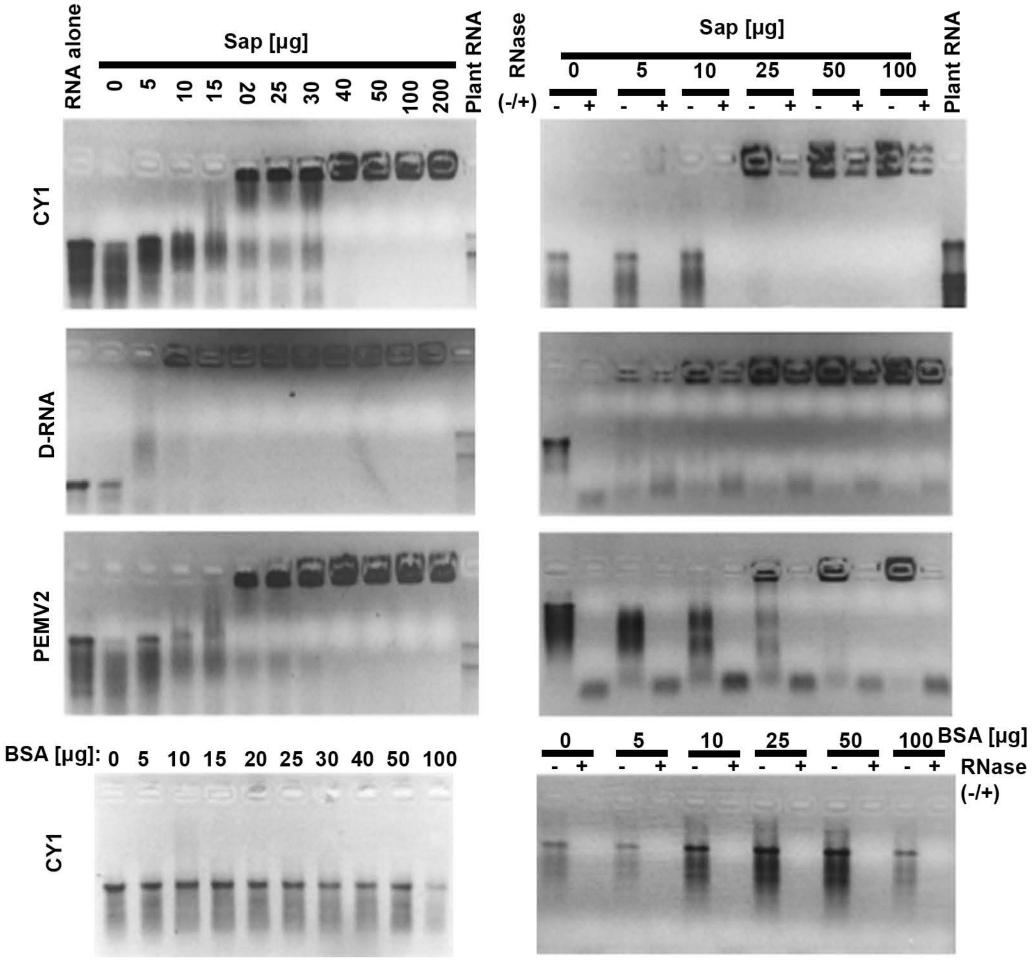

7C_raw_images

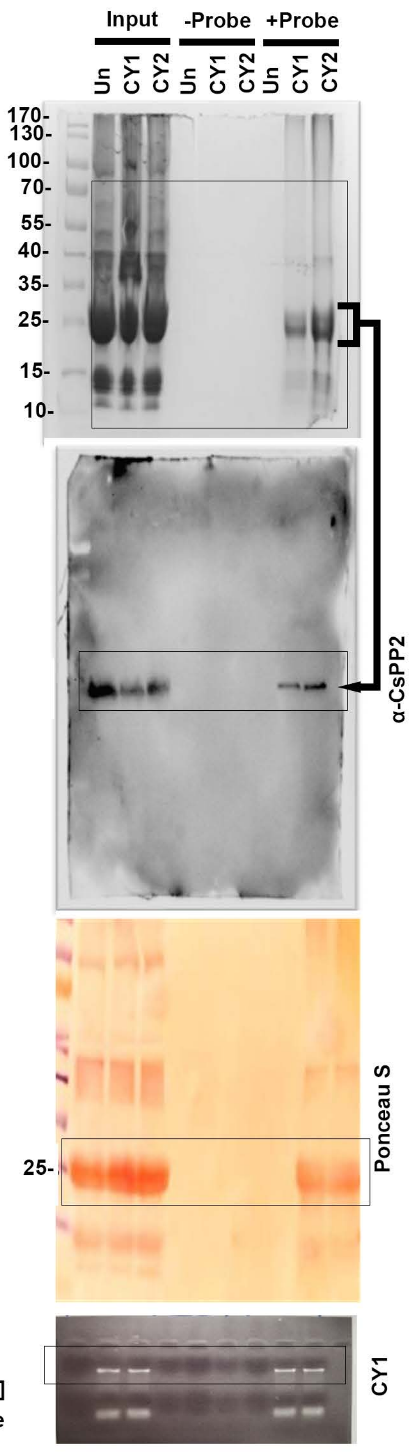

S2B_raw_images

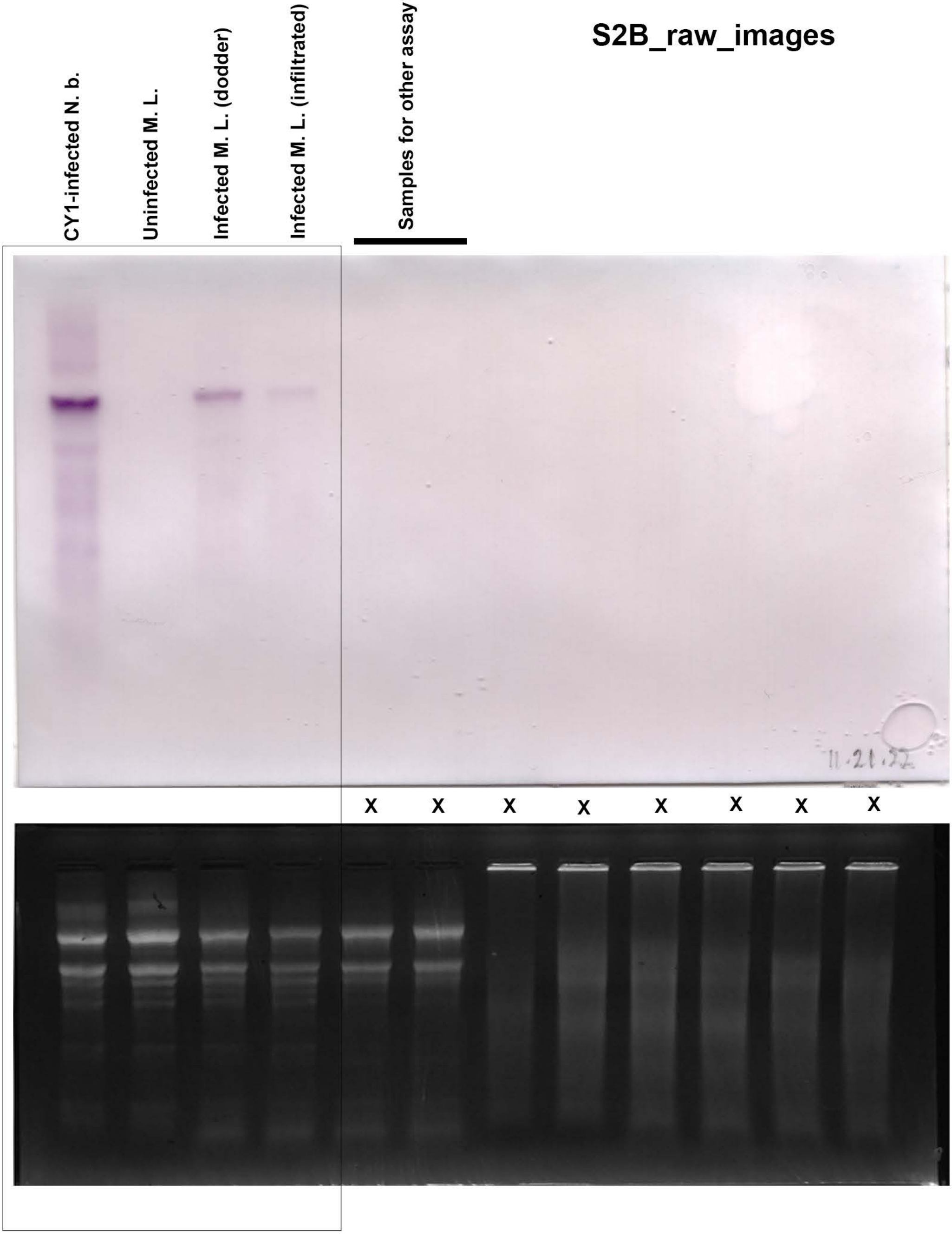

S4_raw_image

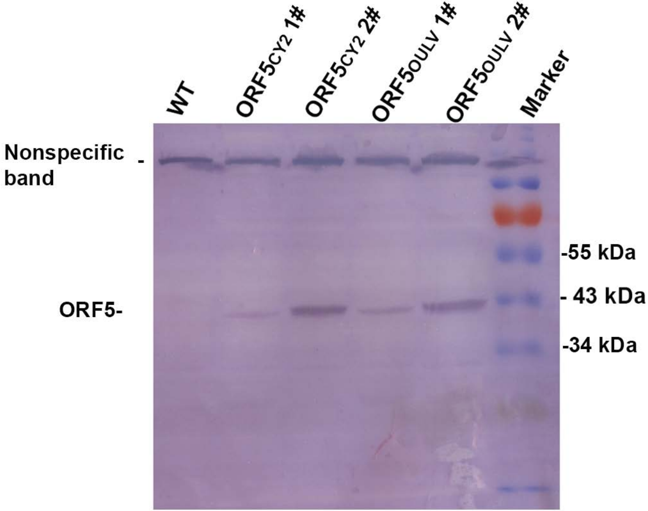
WT
ORF5CY2 1#
ORF5CY2 2#
ORF5OULV 1#
ORF5OULV 2#
Marker
Nonspecific band -
ORF5-
-55 kDa
- 43 kDa
-34 kDa

S8B_raw_images

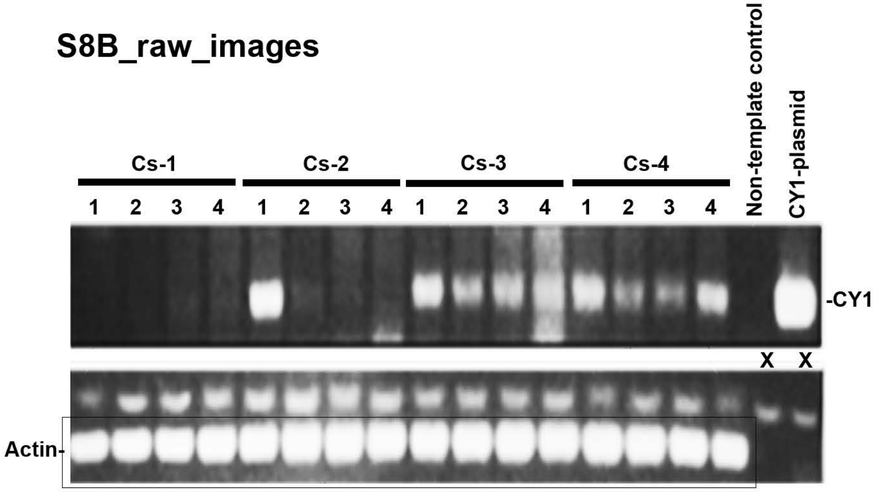

S8C_raw_images

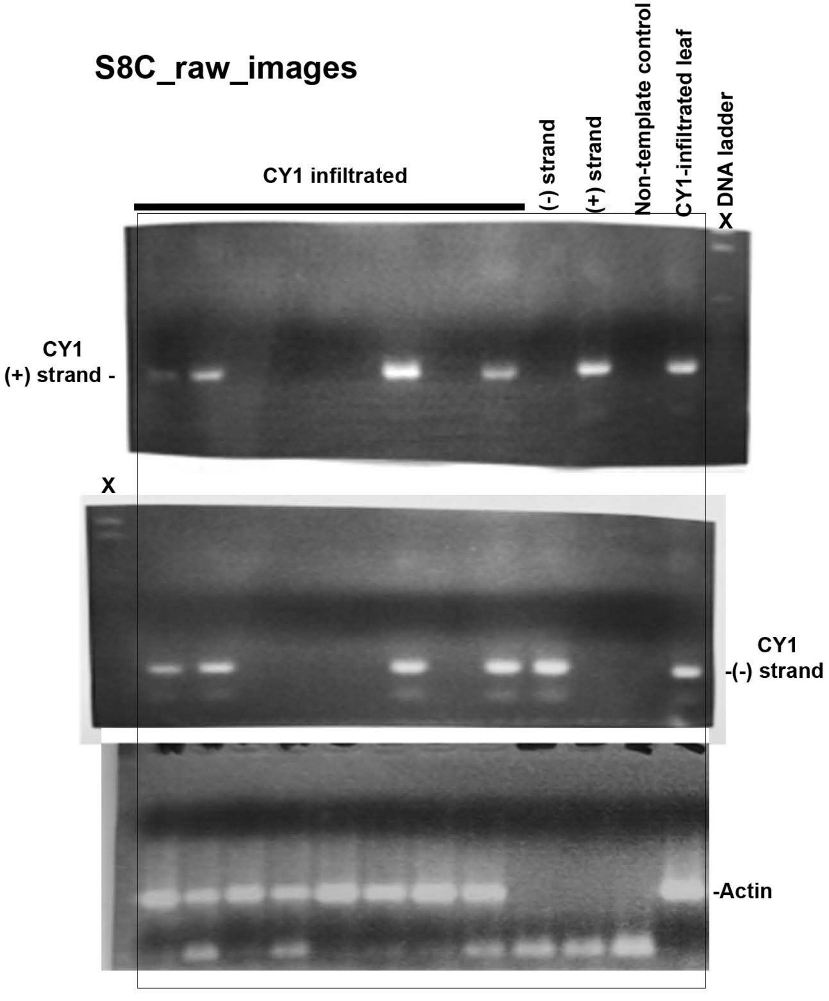

Supplement: S1 Raw Images — (PDF) [file pbio.3002600.s002.pdf]
