# Supplementary material for: Umbravirus-like RNA viruses are capable of independent systemic plant infection in the absence of encoded movement proteins
Source: PLoS Biol. 2024 Apr 25;22(4):e3002600. doi: 10.1371/journal.pbio.3002600 (PMC11081511; doi:10.1371/journal.pbio.3002600)
Supplement: S1 Fig — (A) Genome organization of umbraviruses, Group 1 umbra-like viruses (ULVs), and Group 2/Class 2 ULV CY1. (B) Secondary structure and tertiary interactions either known or predicted to occur at the 3′ terminus of ULVs and umbraviruses. Tertiary interactions are color coded. Conserved residues are in red. PEMV2, umbravirus pea enation mosaic virus; ArULV, arborvitae umbra-like virus (OQ102001); AgULV, Ageratum virus 1 (OP660856); CY1, Citrus yellow vein associated virus 1 (JX101610). (PDF) [file pbio.3002600.s003.pdf]

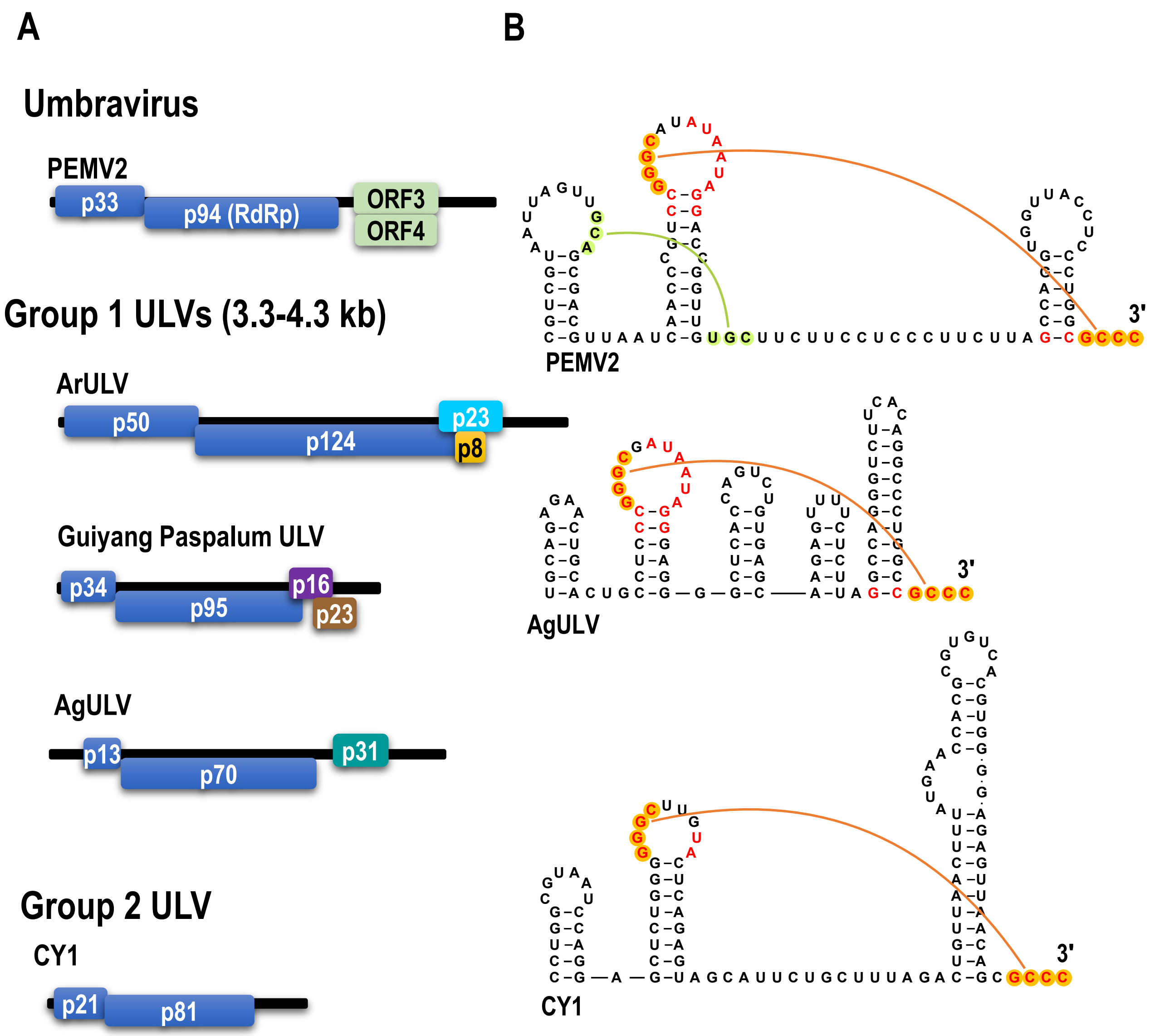

S1 Fig. Umbra-like viruses contain 3' RNA structures similar to umbraviruses. A. Genome organization of umbraviruses, Group 1 umbra-like viruses (ULVs), and Group 2/Class 2 ULV CY1. **B.** Secondary structure and tertiary interactions either known or predicted to occur at the 3' terminus of ULVs and umbraviruses. Tertiary interactions are color coded. Conserved residues are in red. PEMV2, umbravirus pea enation mosaic virus; ArULV, arborvitae umbra-like virus (OQ102001); AgULV, Ageratum virus 1 (OP660856); CY1, Citrus yellow vein associated virus 1 (JX101610).
