# Supplementary material for: Umbravirus-like RNA viruses are capable of independent systemic plant infection in the absence of encoded movement proteins
Source: PLoS Biol. 2024 Apr 25;22(4):e3002600. doi: 10.1371/journal.pbio.3002600 (PMC11081511; doi:10.1371/journal.pbio.3002600)
Supplement: S2 Fig — (A) Two approaches that were used to infect Mexican lime with CY1. Upper panel, two-week-old N. benthamiana were infiltrated with Agrobacterium containing a CY1-expressing Ti plasmid [step 1]. Fourteen days after infiltration, CY1-positive plants were colonized by dodder vines [step 2]. After detection of CY1 in dodder vines by RT-PCR, dodder tips were guided to colonize 2-month-old Mexican lime [step 3]. Lower panel, leaves and stems of 2-month-old Mexican lime were abraded using a Derma microneedle roller (Amazon:B0CH7SHD5T), and then vacuum-infiltrated with Agrobacterium containing a CY1-expressing Ti plasmid. (B) Right, northern blot used to detect CY1 in Mexican lime 15 months after dodder transfer or 12 months after vacuum infiltration. Left, infected plants at these times, and subsequently, did not display discernable systems. N.b., Nicotiana benthamiana; M.L., Mexican lime. (PDF) [file pbio.3002600.s004.pdf]

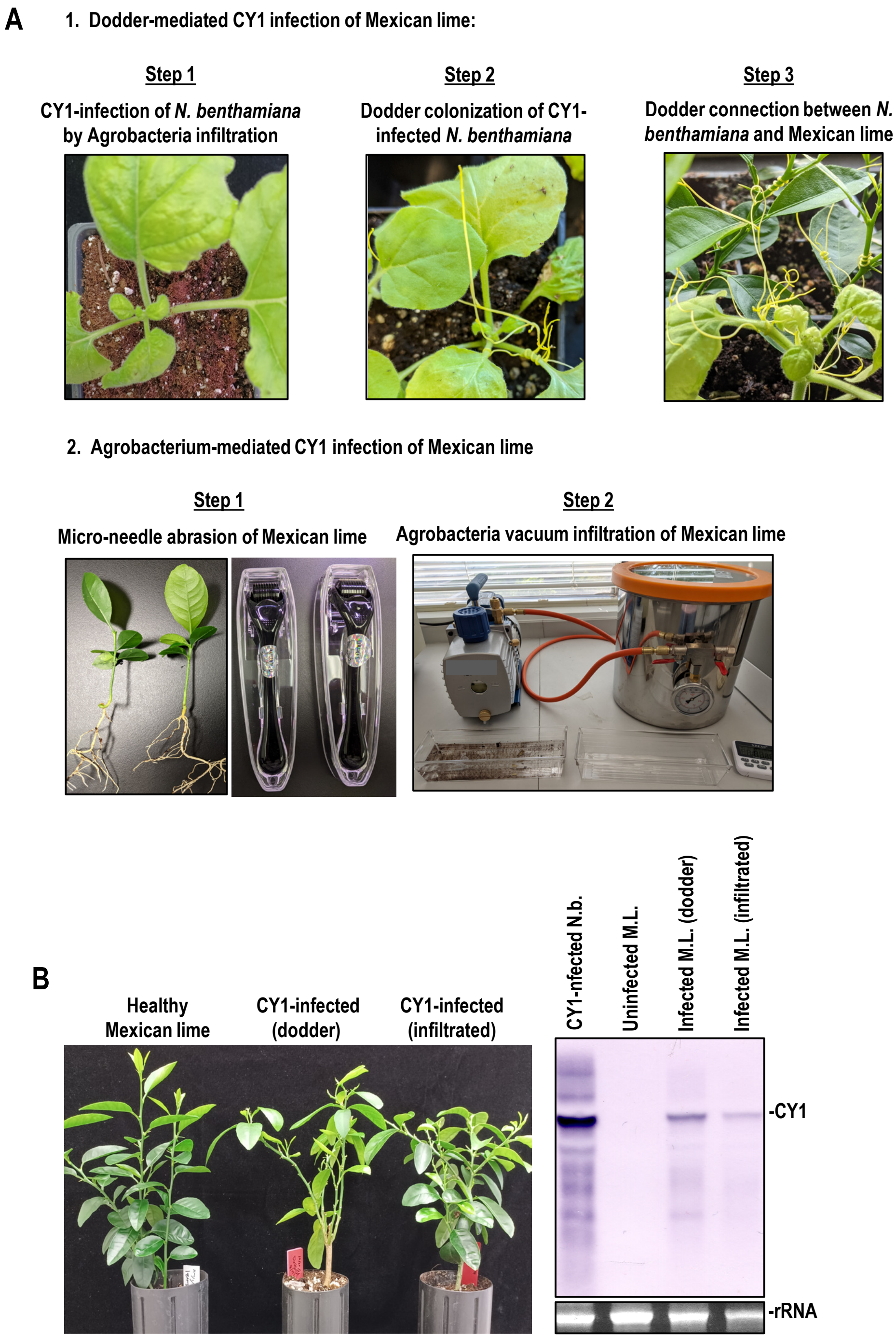

S2 Fig. CY1 systemically infected Mexican lime in the absence of a helper virus. A. Two approaches that were used to infect Mexican lime with CY1. Upper panel, two-week-old *N. benthamiana* were infiltrated with Agrobacterium containing a CY1-expressing Ti plasmid [step 1]. Fourteen days after infiltration, CY1-positive plants were colonized by dodder vines [step 2]. After detection of CY1 in dodder vines by RT-PCR, dodder tips were guided to colonize 2-month-old Mexican lime [step 3]. Lower panel, leaves and stems of 2-month-old Mexican lime were abraded using a Derma microneedle roller (Amazon:B0CH7SHD5T), and then vacuum-infiltrated with Agrobacterium containing a CY1-expressing Ti plasmid. **B.** Right, Northern blot used to detect CY1 in Mexican lime 15-months after dodder transfer or 12-months after vacuum infiltration. Left, infected plants at these times, and subsequently, did not display discernable systems. N.b., *Nicotiana benthamiana*; M.L., Mexican lime.
