# Supplementary material for: Umbravirus-like RNA viruses are capable of independent systemic plant infection in the absence of encoded movement proteins
Source: PLoS Biol. 2024 Apr 25;22(4):e3002600. doi: 10.1371/journal.pbio.3002600 (PMC11081511; doi:10.1371/journal.pbio.3002600)
Supplement: S3 Fig — FISH analysis of CY1, CY2, and CY2sgm1 at 60 dpi. Cy3-labeled oligonucleotide probe targeted CY1 and CY2 positions 1101–1131. DAPI-stained DNA and xylem tissue fluoresce blue. Bar = 50 μm. No viral RNA signals were detected in non-phloem tissue. (PDF) [file pbio.3002600.s005.pdf]

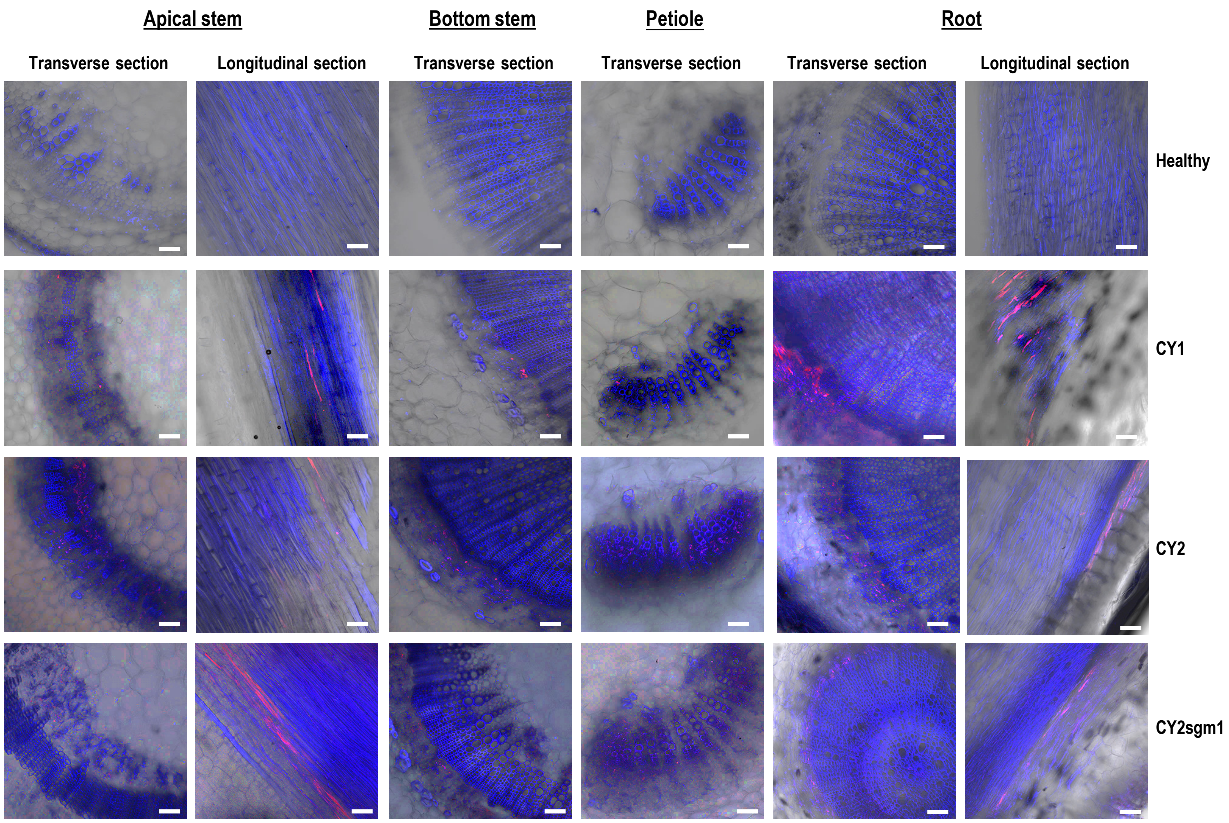

S3 Fig. CY2 and CY2sgm1 accumulated in phloem tissues of stem, petiole, and roots. FISH analysis of CY1, CY2 and CY2sgm1 at 60 dpi**.** Cy3-labeled oligonucleotide probe targeted CY1 and CY2 positions 1101-1131. DAPI-stained DNA and xylem tissue fluoresce blue. Bar = 50 μm. No viral RNA signals were detected in non-phloem tissue.
