# Supplementary material for: Umbravirus-like RNA viruses are capable of independent systemic plant infection in the absence of encoded movement proteins
Source: PLoS Biol. 2024 Apr 25;22(4):e3002600. doi: 10.1371/journal.pbio.3002600 (PMC11081511; doi:10.1371/journal.pbio.3002600)
Supplement: S4 Fig — Western blot detection of ORF5CY2 and ORF5OULV. CY2 is the closest related ORF5-expressing ULV to CY1 and OULV is the least related of the dicot-infecting ULVs. Total proteins were isolated from heterozygous T1 plants and subjected to 10% denaturing PAGE. Primary antibody was developed in rabbits against peptide SLVRETYIPSSTTTGKE. ORF5 proteins are migrating as dimers under the conditions used. (PDF) [file pbio.3002600.s006.pdf]

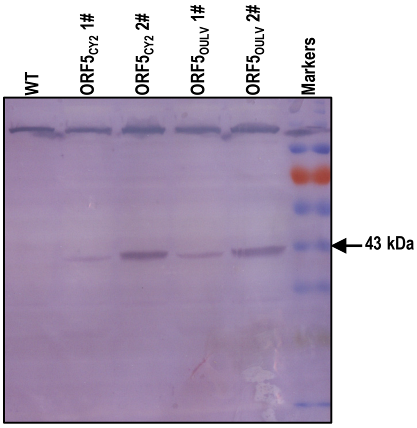

S4 Fig. ***N. benthamiana*** **transgenic plants expressing ORF5 proteins.** Western blot detection of $ORF5_{CY2}$ and $ORF5_{OULV}$. CY2 is the closest related ORF5-expressing ULV to CY1 and OULV is the least related of the dicot-infecting ULVs. Total proteins were isolated from heterozygous T1 plants and subjected to 10% denaturing PAGE. Primary antibody was developed in rabbits against peptide SLVRETYIPSSTTTGKE. ORF5 proteins are migrating as dimers under the conditions used.
