# Supplementary material for: Umbravirus-like RNA viruses are capable of independent systemic plant infection in the absence of encoded movement proteins
Source: PLoS Biol. 2024 Apr 25;22(4):e3002600. doi: 10.1371/journal.pbio.3002600 (PMC11081511; doi:10.1371/journal.pbio.3002600)
Supplement: S5 Fig — (A) Schematic diagram of vectors used to infiltrate N. benthamiana. (B) Representative plants at 14 dpi. Only plants infiltrated with TMV-GFP show GFP expression in systemic leaves. (PDF) [file pbio.3002600.s007.pdf]

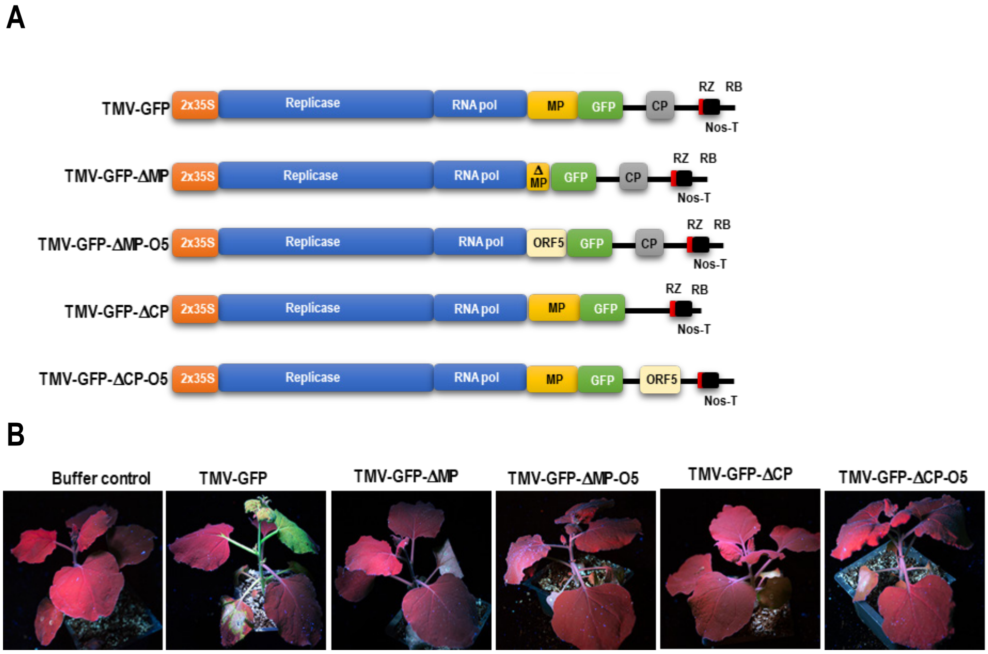

S5 Fig. ORF5$_{CY2}$ does not complement MP-defective TMV-GFP or CP-defective TMV-GFP. A. Schematic diagram of vectors used to infiltrate *N. benthamiana*. **B.** Representative plants at 14 dpi. Only plants infiltrated with TMV-GFP show GFP expression in systemic leaves.
