# Supplementary material for: Umbravirus-like RNA viruses are capable of independent systemic plant infection in the absence of encoded movement proteins
Source: PLoS Biol. 2024 Apr 25;22(4):e3002600. doi: 10.1371/journal.pbio.3002600 (PMC11081511; doi:10.1371/journal.pbio.3002600)
Supplement: S6 Fig — A. NoLS (underlined) at the N-terminal region of ORF5 proteins in Class 2 ULVs. Asterisks denote conserved amino acids. Sequence was predicted using NoLStradamus; http://www.moseslab.csb.utoronto.ca/NoLStradamus/. (B) NoLS (underlined) at the N-terminal region of CPs of viruses from polerovirus (PLRV), luteovirus (BYDV), and sobemovirus (SeMV) genera. (PDF) [file pbio.3002600.s008.pdf]

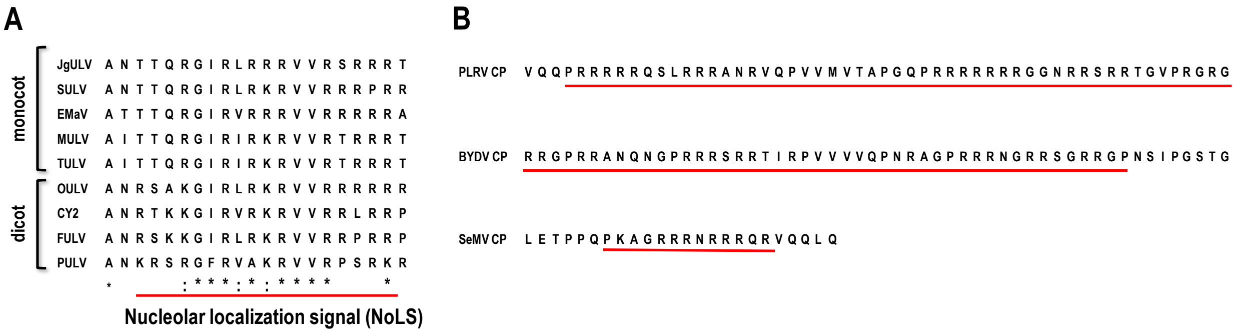

S6 Fig. ORF5 protein contains a NoLS. A. NoLS (underlined) at the N-terminal region of ORF5 proteins in Class 2 ULVs. Asterisks denote conserved amino acids. Sequence was predicted using NoLStradamus; http://www.moseslab.csb.utoronto.ca/NoLStradamus/. **B.** NoLS (underlined) at the N-terminal region of CPs of viruses from polerovirus (PLRV), luteovirus (BYDV), and sobemovirus (SeMV) genera.
