# Supplementary material for: Umbravirus-like RNA viruses are capable of independent systemic plant infection in the absence of encoded movement proteins
Source: PLoS Biol. 2024 Apr 25;22(4):e3002600. doi: 10.1371/journal.pbio.3002600 (PMC11081511; doi:10.1371/journal.pbio.3002600)
Supplement: S7 Fig — (A) Alignment of ORF5 proteins with selected 30K MPs. Critical 30K MP aspartic acid residue (D) is in red. Bottom row denotes the consensus secondary structure predicted in PROMALS3D where e represents a β-strand. (B) Charge distribution for ORF5CY2, CPBYDV, and MPOuLV by amino acid residue position (window size = 5) analyzed in EMBOSS CHARGE version 6.6.0. Red box denotes the jelly-roll domain. Note that charged residues are concentrated in the N-terminal region of ORF5CY2 and CPBYDV but not MPOuLV. (PDF) [file pbio.3002600.s009.pdf]

A

OuMV 30K MP	L - A R G Q I A V V **D** T R V - - -
SeMV CP	T - - A G S I H M G F Q Y D M - A
PULV	T - - Q - - I V G I G N A P S K D
JgULV	T - - Q - - I V G L G N C H S S V
TULV	T - - Q - - I V G L A N A D G - A
MULV	T - - Q - - I V G L A N A D G - A
EMaV	T - - Q - - I A G V G N A D S F Q
SULV	T - - Q - - I C G V G N A D Y Y T
EMaV	T - - Q - - I A G V G N A D S Y Q
FULV	T - - Q - - H C G V G L S S S G A
CY2	T - - Q - - L C G V G L S T S G A
OULV	T - - Q - - H C G V G L A T S G A
PEMV2 30K MP	V L G E V E I W L H **D** S I L P H L
CMoV 30K MP	S P G Q A V I W V H **D** T A L P G L
TMV 30K MP	C R G G V S V C L V **D** K R M E R A
Consensus	e e e e e

B

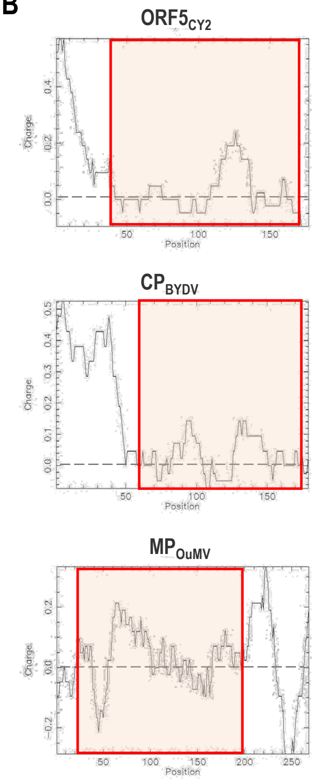

S7 Fig. ORF5 proteins have features inconsistent with canonical 30K MPs. A. Alignment of ORF5 proteins with selected 30K MPs. Critical 30K MP aspartic acid residue (D) is in red. Bottom row denotes the consensus secondary structure predicted in PROMALS3D where e represents a β-strand. **B.** Charge distribution for $ORF5_{CY2}$, CP_{BYDV}, and MP_{OuLV} by amino acid residue position (window size = 5) analyzed in EMBOSS CHARGE version 6.6.0. Red box denotes the jelly-roll domain. Note that charged residues are concentrated in the N-terminal region of $ORF5_{CY2}$ and CP_{BYDV} but not MP_{OuLV}.
