# Supplementary material for: Umbravirus-like RNA viruses are capable of independent systemic plant infection in the absence of encoded movement proteins
Source: PLoS Biol. 2024 Apr 25;22(4):e3002600. doi: 10.1371/journal.pbio.3002600 (PMC11081511; doi:10.1371/journal.pbio.3002600)
Supplement: S8 Fig — (A) Schematic representation of a cucumber plant (Cucumis sativus cv. Spacemaster) agroinfiltrated with CY1 on cotyledons (solid green). Systemic leaves (numbered 1 to 4) were used to test for the presence of CY1. (B) Representative RT-PCR detection of CY1 in systemic leaves of 4 cucumber plants (Cs-1 to Cs-4) at 14 dpi. Negative control was the reaction in the absence of template and positive control was plasmid DNA containing full-length CY1. (C) Strand-specific detection of (+)- and (-)-strand CY1 in systemic leaves of 8 cucumber plants infiltrated with CY1 at 21 dpi. (-)-strand and (+)-strand lanes used strand-specific primers and plasmid DNA containing full-length CY1. (PDF) [file pbio.3002600.s010.pdf]

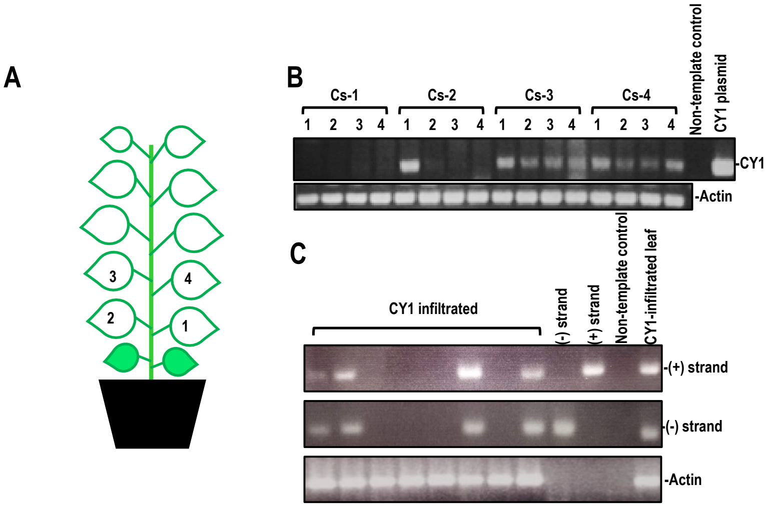

S8 Fig. CY1 systemically infects cucumber. A. Schematic representation of a cucumber plant (*Cucumis sativus* cv. Spacemaster) agroinfiltrated with CY1 on cotyledons (solid green). Systemic leaves (numbered 1 to 4) were used to test for the presence of CY1. **B.** Representative RT-PCR detection of CY1 in systemic leaves of 4 cucumber plants (Cs-1 to Cs-4) at 14 dpi. Negative control was the reaction in the absence of template and positive control was plasmid DNA containing full-length CY1. **C.** Strand-specific detection of (+)- and (-)-strand CY1 in systemic leaves of 8 cucumber plants infiltrated with CY1 at 21 dpi. (-)-strand and (+)-strand lanes used strand-specific primers and plasmid DNA containing full-length CY1.
