# Supplementary material for: Umbravirus-like RNA viruses are capable of independent systemic plant infection in the absence of encoded movement proteins
Source: PLoS Biol. 2024 Apr 25;22(4):e3002600. doi: 10.1371/journal.pbio.3002600 (PMC11081511; doi:10.1371/journal.pbio.3002600)
Supplement: S9 Fig — (PDF) [file pbio.3002600.s011.pdf]

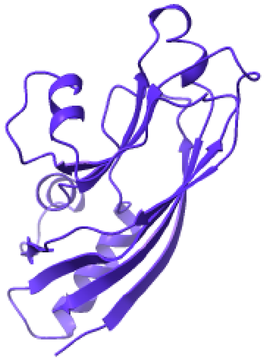

MP$_{OuMV}$

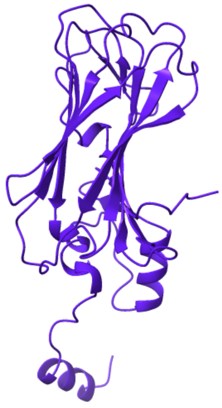

PP2 (*C. moschata*)
(UniProtKB: Q9LLT3)

S9 Fig. Structural similarity between the central domains of the ourmia melon virus MP (MP$_{OuMV}$) and cucumber PP2.
