# Supplementary material for: Umbravirus-like RNA viruses are capable of independent systemic plant infection in the absence of encoded movement proteins
Source: PLoS Biol. 2024 Apr 25;22(4):e3002600. doi: 10.1371/journal.pbio.3002600 (PMC11081511; doi:10.1371/journal.pbio.3002600)
Supplement: S10 Fig — Photos of 4 plants each infected with WT CY1, CY1-random, and CY1-PP2 were taken at 50 dpi. (PDF) [file pbio.3002600.s012.pdf]

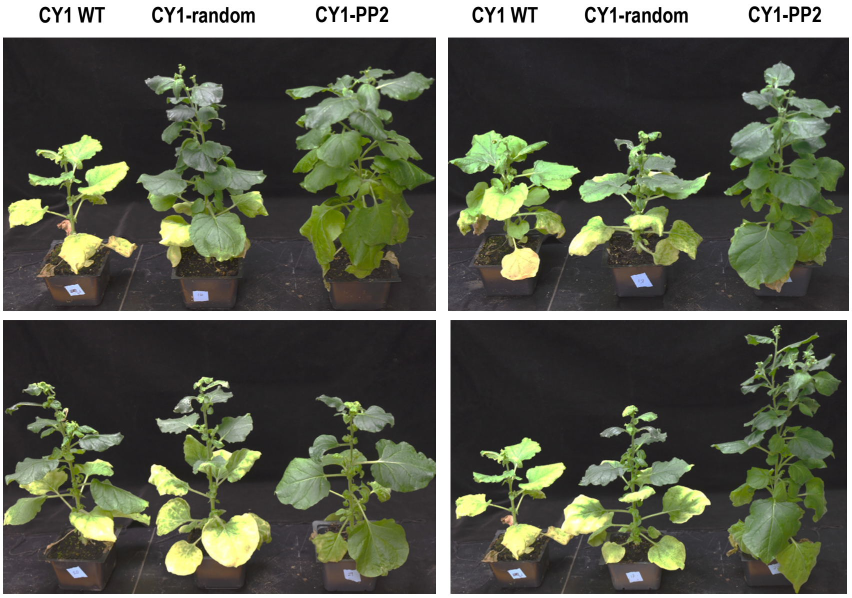

S10 Fig. CY1-mediated silencing of *PP2* mRNA results in less severe symptoms on *N. benthamiana*. Photos of 4 plants each infected with WT CY1, CY1-random, and CY1-PP2 were taken at 50 dpi.
