# Supplementary material for: Umbravirus-like RNA viruses are capable of independent systemic plant infection in the absence of encoded movement proteins
Source: PLoS Biol. 2024 Apr 25;22(4):e3002600. doi: 10.1371/journal.pbio.3002600 (PMC11081511; doi:10.1371/journal.pbio.3002600)
Supplement: Attachment — Submitted filename: Response to reviewers.pdf [file pbio.3002600.s013.pdf]

Response to reviewers

Rev. 1:
The manuscript entitled with "Independent systemic infection of umbravirus-like RNA viruses in the absence of an encoded movement protein" by Ying et al reported their findings on umbravirus-like RNA viruses (ULVs) that do not encode movement protein (MP), but could systemically infect Nicotiana benthamiana in a phloem-limited fashion when delivered by agroinfiltration. The authors generated full-length clones of CY1 and CY2 and examined their abilities to systemically infect the model plant N. benthamiana. CY1 encodes replication proteins only, but CY2 closely related CY1, which encodes an additional protein (ORF5) that has characteristics more similar to luteoviruses/poleroviruses/sobemovirus capsid proteins than to 30K MPs and ORF5 was dispensable for infection of CY2, but was associated with faster symptom development. In systemically infected CY2-infected plants, small virus-like particles (VLPs) were identified, suggesting that CY2 uses ORF5 as a CP, which would explain the absence of an umbravirus-type helper virus in a sample from hemp containing CY2.

When CY1 was used as a virus-induced gene silencing (VIGS) vector to reduce PP2 transcripts, a phloem protein 2 (PP2), an enigmatic, dimeric, chitin-binding lectin encoded by a large gene family is highly abundant in phloem sap and is considered to be a non-specific RNA-binding protein, in which CY1 accumulation was reduced in systemic leaves, supporting the possibility that PP2 is the MP for CY1.

The story is interesting and the paper is well written. Before considering for acceptance publication, there are some points below that need to be addressed and corrected before

Major concerns:
1. Does reducing PP2 expression also affects CY2 accumulation and systemic movement?

CY1 and CY2 are very closely related, although CY1 has lost the ability to synthesize ORF5 protein. As we showed, when CY2 is mutated to no longer produce the ORF5 protein, the timing to first infection is identical to CY1 and symptoms are identical and phloem restriction is identical. This strongly suggests that CY1 and CY2 (and likely all of the Group 2/Class 2 ULA RNAs) are using PP2.

Unfortunately, the presence of ORF5 precludes the usage of CY2 as a VIGS vector unless we eliminate expression of ORF5, which defeats the purpose of the experiment.

Minor:
1. Line 39 and 145: it should be clear which protein from CY1 interacts with PP2.

It's the CY1 RNA that interacts with PP2. To eliminate the confusion, we have added "RNA" after CY1.

Rev. 2:
Ying et al. report the first plant virus (CY) that establishes a systemic infection with no need of a movement protein (MP). This finding challenges the dogma that currently known plant viruses need of this protein to colonize their host. Therefore, this work represents a ground-breaking advance in our understanding of plant-virus interactions. Owing to the

relevance of the subject and conclusions of the manuscript, the authors not only demonstrate that CY1, which only encodes replication proteins, systemically colonizes different hosts, but also demonstrate that CY2, which encodes a protein potentially acting as a MP (ORF5) do the same even in the absence of ORF5, which is more likely a coat protein. Finally, the authors provide an explanation on how these viruses manage to establish systemic infections by showing that they appear to interact with host proteins with similar activities than viral MPs such as PP2. The manuscript is very well written, experiments are presented
in a logical manner and with a clear connection and rationale linking them, and results support the conclusions of the paper. Overall, I find this to be a notable contribution to PLoS Biology and I only have some comments on aspects that need to be clarify but won´t change the conclusions of the work. In order of appearance:

222-225. Table 1. I had some difficulties to understand the statistical analysis here. It seems that numbers reflect percentages of symptomatic plants over 45-50 individuals assayed. Then, how did the authors perform a Student´s t-test? What was considered a replicate here? Would not be more logical just to apply a Fisher exact test, which is more appropriate for comparing frequencies? Conclusions are unlikely to change, but I would much appreciate if the authors could clarify this point.

Table 1 displays the total number of plants that were infiltrated and the percentage of plants that showed symptoms at various time points across three independent experiments. To simplify, we combined the data. For statistical analysis, each repetition is considered one replicate. Therefore, we obtained the means for different viral infiltrations per time point from three replicates. A Student's t-test was performed to obtain the significance of each pair of mean comparisons within a single time point.

As for the suggestion to use Fisher's exact test, but it cannot be performed in this case since there is only one categorical variable for each time point, that being viral infiltrations. Fisher's exact test is typically used to test the association between two categorical variables. Therefore, for each time point (e.g. 6 wpi), we checked if there was a significant difference between the number of plants showing symptoms infiltrated with different viral strains. We have reworded the legend below the table for clarity as follows:

These results are from three independent experiments. For statistical analysis, independent experiments were considered replicates to calculate means that were further subjected to Student’s t, $\alpha = 0.05$, for pairwise comparisons. Means with the same letter(s) are not significantly different within a column. wpi, weeks-post infiltration

228-235 and Figure 4. The authors state that CY2sgm1 and 2 mutants do not express the subgenomic RNA encoding ORF5 and this is supported by data on Figure 4B. However, I am intrigued by data on Figure 4C which, in the middle panel, shows a band of seemingly the same size of the subgenomic RNA for lines of single infections by CY2sgm1. Is it possible that small quantities of subgenomic RNA would be expressing?

CYVaV infection from transcripts always eventually produces a very specific defective RNA (D-RNA; 924 nt) that probes also bind, which is similar in size to the sgRNA (831 nt) observed in the middle panel. It is possible that the faint band is this D-RNA or it could be very low levels of the sgRNA. Since this RNA band was not detected in protoplasts, the band was likely the D-

RNA. This information has been added to the figure legend as follows: ●, denotes low level band that is not detected in CY2sgm1 protoplasts infections that is near the size of the sgRNA (831 nt) and also the size of a defective (D)-RNA (924 nt) that commonly arises in CY1/CY2 infections.

333-342. Detection of VLPs point to ORF5 as a CP involved in virion formation. However, it might be well possible that these are empty virions that can self-assemble as has been shown for other plant viruses. I understand that demonstrating that the detected VLPs are infectious/transmissible is out of the scope of the work. However, did the authors at least try to purify these particles and to check if viral RNA could be detected by disassembling them?

Currently, we do not know if the virions are mainly empty or have encapsidated gRNA. CY2 RNA and the sgRNA were detected associated with the semi-purified virions, so it is likely that they are not all empty capsids but we did not further purify the virions for this report. Purification of virions and cryo-EM will be conducted in the near future.

390-403 and Figure 8. If I understood correctly, the random hairpin acts as a negative control both to check that only the PP2 hairpin affects PP2 expression and that introducing the hairpin does not affect the outcome of infection as compared with the WT. Although the statistical test is not shown, looking at figure 8 I assume that CY1-WT and CY1-random induce similar levels of PP2 expression as expected. Thus, one would expect to see similar levels of virus accumulation in WT and random CY1s. However, panel C shows that CY1-random accumulates at higher levels than the WT. This could suggest that the random hairpin is somehow affecting the progress of the infection. Did the authors consider this possibility?

The reviewer is correct that both CY1-WT and CY1-random induce similar levels of PP2 expression, as expected ($P = 0.392$). The random hairpin serves as a negative control to verify that the introduction of the hairpin does not negatively affect the outcome of infection compared to WT. We have made many inserts into CY1, and occasionally get increased accumulation (mostly get decreased accumulation).

On the same figure, in panel B most replicates of CY1-random show higher PP2 expression that replicates of CY1-PP2. Oddly, in panel C there is at least one CY1-random replicate in which virus accumulation is 0. Does this mean that PP2 can be over-expressed even in the absence of the virus? How this would affect the authors conclusions?

One of the CY2-random plants didn't infect. Our infection rate is close to 100% but It's not 100%. This would be analogous, therefore to the "empty vector" since Agrobacteria, like most (all) phytobacteria likely induce expression of PP2. Several of the PP2 level data points in the CY1-random are similar to the empty vector, so it is internally consistent (but I had to think about it too!).

Finally, and I apologize for repeating myself, it is unclear why in these analyses a different statistical test is used. First, the LSD (which is a post-hoc test) is presented before the main test, which I understand is the Omnibus test. Second, why using here the omnibus test instead the Student´s t-test? Perhaps it would be worth adding a section on the Material and Methods explaining the statistical methodology.

We appreciate the reviewer for pointing this out. Yes, the Omnibus test should have been mentioned before the LSD and has been corrected now. As the data was neither normally distributed nor homoscedastic, parametric tests like Student's t-test could not be applied. Thus,

the data was analyzed using Generalized linear models (GLMs). We have updated the figure legend with information on the statistical methodology, which is in bold.

Fig 8. CY1 VIGS vector targeting mRNA of two PP2 family members decreases CY1 accumulation in systemic leaves. A. Hairpin used to target the two PP2 mRNAs and similarly sized random hairpin. Hairpins replaced the natural hairpin at position 2220. Agrobacteria constructs harboring either PEMV2, CY1 WT, CY1-PP2 or CY1-random were agroinfiltrated into N. benthamiana leaves. Total RNA from systemic leaves was extracted for relative quantification of RNA at 28-dpi. B. PP2- specific primers were used for quantification of PP2 relative to actin. C. CY1-specific primers were used for quantification of CY1. Data were analyzed using generalized linear models (GLMs) as the data were neither normally distributed nor homoscedastic. The omnibus test (likelihood ratio chi-square test) gave a $p \leq 0.012$, rejecting the null hypothesis that treatments did not have any effect on accumulation. Significance was determined using differences at a *p*-value of * < 0.05, ** < 0.1, and *** < 0.001 as determined from a least significance difference (LSD) test. Error bars represent standard error. Experiments were performed with n > 6 biological replicates per treatment."

461. In several parts of the manuscript a defective RNA is used and mentioned to be described elsewhere, but I found no description of it in the text. If the authors meant that it will be described in a different article, perhaps it would be worth asking if the analyses using the defective RNA are central to make their point, and if the answer is "no" I would recommend removing them. If the authors chose to maintain the analyses with the defective RNA, I think it would be necessary to add at least a brief description of it to facilitate the reader to understand the results.

We would like to keep the D-RNA in this figure as it is most clearly protected from RNase treatment. The reviewer is correct about the additional information and we have added the following to the abstract:

CY1 RNA and a defective (D)-RNA that arises during infection...

And also added the following explanation to the text:

Northwestern assays were performed using cucumber phloem sap and uniformly labeled (*i*) CY1; (*ii*) a CY1 defective RNA (D-RNA; 921 nt) composed of positions 1-671 joined to positions 2442-2693 that is frequently found in CY1- and CY2-infected *N. benthamiana*;

490-495. I had some problems following this part of the text. Do the authors mean that part of the CY2 clone was generated using CY1 as a template because the CY2 sequence was unknown? Would not this make a chimera? To which extent could this affect the authors conclusions?

The complete CY2 sequence was synthesized by Twist Bioscience company based on the sequence (accession number MT893741) from NCBI. However, the assembled sequence (MT893741) is not complete and was poorly annotated. The NCBI sequence had 3' end sequence at its 5' end, which was used but was missing 16 nt. We used CY1 sequence for these 16 nt. Since this time, we have generated a full-length CY2 construct from a hemp field sample and its 16 nt were identical to those found in this CY2 sequence. Therefore, we are confident in our conclusions and do not consider our CY2 to be a chimera.

As a general minor comment, I suggest the authors to revise scientific names of plant species as they are not consistently written in italics.

This seems to be a journal-specific issue. We normally do not italicize plant species if the name is used as an adjective. The copy editors can correct according to the journal standards.
