# Supplementary material for: Umbravirus-like RNA viruses are capable of independent systemic plant infection in the absence of encoded movement proteins
Source: PLoS Biol. 2024 Apr 25;22(4):e3002600. doi: 10.1371/journal.pbio.3002600 (PMC11081511; doi:10.1371/journal.pbio.3002600)
Supplement: Attachment — Submitted filename: Second response to the editor.pdf [file pbio.3002600.s014.pdf]

DATA POLICY: IMPORTANT - PLEASE READ

You may be aware of the PLOS Data Policy, which requires that all data be made available without restriction: http://journals.plos.org/plosbiology/s/data-availability. For more information, please also see this editorial: http://dx.doi.org/10.1371/journal.pbio.1001797

Request for a title change to: **Umbravirus-like RNA viruses are capable of systemic plant infection in the absence of movement proteins**

We have used the new title with the exception that we have added "encoded" and "independent"

Umbravirus-like RNA viruses are capable of independent systemic plant infection in the absence of encoded movement proteins

The viruses certainly use movement proteins- just host movement proteins and not encoded movement proteins (the very first plant viruses without encoded movement proteins). This is now clear in the new title.

Also, with your suggested title, it isn't clear that these viruses don't just require a helper virus like umbraviruses do. It is important that the title conveys that the infection is "independent" (of any helper virus). Hopefully this new title is acceptable.

Note that we do not require all raw data. Rather, we ask that all individual quantitative observations that underlie the data summarized in the figures and results of your paper be made available in one of the following forms:

1) Supplementary files (e.g., excel). Please ensure that all data files are uploaded as 'Supporting Information' and are invariably referred to (in the manuscript, figure legends, and the Description field when uploading your files) using the following format verbatim: S1 Data, S2 Data, etc. Multiple panels of a single or even several figures can be included as multiple sheets in one excel file that is saved using exactly the following convention: S1_Data.xlsx (using an underscore).

S2 data (for Fig 8) is now uploaded into supporting information, and this sentence has been added to the figure legend that is associated with Fig. 8. A new version of Fig. 8 was uploaded.

"Source data can be found in S2 Data in Supporting Information".

2) Deposition in a publicly available repository. Please also provide the accession code or a reviewer link so that we may view your data before publication.

Raw images have been deposited at: **https://figshare.com/account/articles/25407199**

A statement to this effect was added under "Supporting Information"

Regardless of the method selected, please ensure that you provide the individual numerical values that underlie the summary data displayed in the following figure panels as they are essential for readers to assess your analysis and to reproduce it: Fig. 8B, C

S1 data (for Fig 8) is now uploaded into supporting information, and this sentence has been added to the figure legend that is associated with Fig. 8. A new version of Fig. 8 was uploaded.

“Source data can be found in S2 Data in Supporting Information”.

NOTE: the numerical data provided should include all replicates AND the way in which the plotted mean and errors were derived (it should not present only the mean/average values).

Please also ensure that figure legends in your manuscript include information on WHERE THE UNDERLYING DATA CAN BE FOUND, and ensure your supplemental data file/s has a legend.

Hopefully what is presented in S2 Data is what you are looking for.

Please ensure that your Data Statement in the submission system accurately describes where your data can be found.

BLOT AND GEL REPORTING REQUIREMENTS:

Many thanks for providing the original, uncropped gels. I have checked the files and I am missing the original images for the following files, thus please upload a new file including these:

Fig. S2B, Fig. S4 and Fig. S8B, C

The file that is uploaded to Figshare now includes the missing gels for these supplemental figures.

DATA NOT SHOWN or UNPUBLISHED

- Please note that per journal policy, we do not allow the mention of "data not shown", "personal communication", "manuscript in preparation" or other references to data that is not publicly available or contained within this manuscript. Please either remove the mention of these data in line 419 or provide figures presenting the results.

That sentence has been deleted
