# Supplementary material for: Umbravirus-like RNA viruses are capable of independent systemic plant infection in the absence of encoded movement proteins
Source: PLoS Biol. 2024 Apr 25;22(4):e3002600. doi: 10.1371/journal.pbio.3002600 (PMC11081511; doi:10.1371/journal.pbio.3002600)
Supplement: Peer review history

# Editor report: Decision Letter 0

**DOI:** 10.1371/journal.pbio.3002600.r001

**Authors:** Paula Jauregui, PhD

7 Nov 2023

Dear Dr Simon, 

Thank you for submitting your manuscript entitled "Independent systemic infection of umbravirus-like RNA viruses in the absence of an encoded movement protein" for consideration as a Research Article by PLOS Biology.

Your manuscript has now been evaluated by the PLOS Biology editorial staff as well as by an academic editor with relevant expertise and I am writing to let you know that we would like to send your submission out for external peer review.

However, before we can send your manuscript to reviewers, we need you to complete your submission by providing the metadata that is required for full assessment. To this end, please login to Editorial Manager where you will find the paper in the 'Submissions Needing Revisions' folder on your homepage. Please click 'Revise Submission' from the Action Links and complete all additional questions in the submission questionnaire.

Once your full submission is complete, your paper will undergo a series of checks in preparation for peer review. After your manuscript has passed the checks it will be sent out for review. To provide the metadata for your submission, please Login to Editorial Manager (https://www.editorialmanager.com/pbiology) within two working days, i.e. by Nov 09 2023 11:59PM.

If your manuscript has been previously peer-reviewed at another journal, PLOS Biology is willing to work with those reviews in order to avoid re-starting the process. Submission of the previous reviews is entirely optional and our ability to use them effectively will depend on the willingness of the previous journal to confirm the content of the reports and share the reviewer identities. Please note that we reserve the right to invite additional reviewers if we consider that additional/independent reviewers are needed, although we aim to avoid this as far as possible. In our experience, working with previous reviews does save time. 

If you would like us to consider previous reviewer reports, please edit your cover letter to let us know and include the name of the journal where the work was previously considered and the manuscript ID it was given. In addition, please upload a response to the reviews as a 'Prior Peer Review' file type, which should include the reports in full and a point-by-point reply detailing how you have or plan to address the reviewers' concerns. 

During the process of completing your manuscript submission, you will be invited to opt-in to posting your pre-review manuscript as a bioRxiv preprint. Visit http://journals.plos.org/plosbiology/s/preprints for full details. If you consent to posting your current manuscript as a preprint, please upload a single Preprint PDF. 

Feel free to email us at plosbiology@plos.org if you have any queries relating to your submission.

Kind regards,

Nonia

Nonia Pariente, PhD 

Editor in Chief

PLOS Biology

npariente@plos.org

for 

Paula Jauregui, PhD 

Editor

PLOS Biology

pjaureguionieva@plos.org

# Peer review: Decision Letter 1

**DOI:** 10.1371/journal.pbio.3002600.r002

**Authors:** Ines Alvarez-Garcia

9 Feb 2024

Dear Dr Simon,

Thank you for your patience while your manuscript entitled "Independent systemic infection of umbravirus-like RNA viruses in the absence of an encoded movement protein" went through peer-review at PLOS Biology. Please accept again our sincere apologies for the time it has taken us to provide you with a decision. Your manuscript has now been evaluated by the PLOS Biology editors, an Academic Editor with relevant expertise, and by two independent reviewers.

As you will see, the reviewers find the results novel and interesting, but they also raise several issues that should be clarified. Reviewer 1 asks whether or not reducing PP2 expression also affects CY2 accumulation and systemic movement, whereas Reviewer 2 has some questions about the statistics used in some of the experiments and several clarifications.

In light of the reviews, we are pleased to offer you the opportunity to address the comments from the reviewers in a revision that we anticipate should not take you very long. We will then assess your revised manuscript and your response to the reviewers' comments with our Academic Editor aiming to avoid further rounds of peer-review, although might need to consult with the reviewers, depending on the nature of the revisions.

We expect to receive your revised manuscript within 1 month. Please email us (plosbiology@plos.org) if you have any questions or concerns, or would like to request an extension. 

At this stage, your manuscript remains formally under active consideration at our journal; please notify us by email if you do not intend to submit a revision so that we withdraw the manuscript.

**IMPORTANT - SUBMITTING YOUR REVISION**

Your revisions should address the specific points made by each reviewer. Please submit the following files along with your revised manuscript:

1. A 'Response to Reviewers' file - this should detail your responses to the editorial requests, present a point-by-point response to all of the reviewers' comments, and indicate the changes made to the manuscript. 

*NOTE: In your point-by-point response to the reviewers, please provide the full context of each review. Do not selectively quote paragraphs or sentences to reply to. The entire set of reviewer comments should be present in full and each specific point should be responded to individually.

You should also cite any additional relevant literature that has been published since the original submission and mention any additional citations in your response. 

2. In addition to a clean copy of the manuscript, please also upload a 'track-changes' version of your manuscript that specifies the edits made. This should be uploaded as a "Revised Article with Changes Highlighted " file type. 

3. Resubmission Checklist

When you are ready to resubmit your revised manuscript, please refer to this resubmission checklist: https://plos.io/Biology_Checklist

To submit a revised version of your manuscript, please go to https://www.editorialmanager.com/pbiology/ and log in as an Author. Click the link labelled 'Submissions Needing Revision' where you will find your submission record. 

Please make sure to read the following important policies and guidelines while preparing your revision and fulfil the editorial requests:

a) *PLOS Data Policy*

Please note that as a condition of publication PLOS' data policy (http://journals.plos.org/plosbiology/s/data-availability) requires that you make available all data used to draw the conclusions arrived at in your manuscript. If you have not already done so, you must include any data used in your manuscript either in appropriate repositories, within the body of the manuscript, or as supporting information (N.B. this includes any numerical values that were used to generate graphs, histograms etc.). Please also indicate in each figure legend where the data can be found. For an example see here: http://www.plosbiology.org/article/info%3Adoi%2F10.1371%2Fjournal.pbio.1001908#s5

b) *Published Peer Review*

Please note while forming your response, if your article is accepted, you may have the opportunity to make the peer review history publicly available. The record will include editor decision letters (with reviews) and your responses to reviewer comments. If eligible, we will contact you to opt in or out. Please see here for more details:

https://blogs.plos.org/plos/2019/05/plos-journals-now-open-for-published-peer-review/

c) *Blot and Gel Data Policy*

Please provide the original, uncropped and minimally adjusted images supporting all blot and gel results reported in an article's figures or Supporting Information files. We will require these files before a manuscript can be accepted so please prepare them now, if you have not already uploaded them. Please carefully read our guidelines for how to prepare and upload this data: https://journals.plos.org/plosbiology/s/figures#loc-blot-and-gel-reporting-requirements

d) *Protocols deposition*

To enhance the reproducibility of your results, we recommend that if applicable you deposit your laboratory protocols in protocols.io, where a protocol can be assigned its own identifier (DOI) such that it can be cited independently in the future. For instructions see: https://journals.plos.org/plosbiology/s/submission-guidelines#loc-materials-and-methods

Thank you again for your submission to our journal. We hope that our editorial process has been constructive thus far, and we welcome your feedback at any time. Please don't hesitate to contact us if you have any questions or comments.

Sincerely,

Ines

--

Ines Alvarez-Garcia, PhD

Senior Editor

PLOS Biology

ialvarez-garcia@plos.org

----------------------------------------------------------------

Reviewers' comments

Rev. 1:

The manuscript entitled with "Independent systemic infection of umbravirus-like RNA viruses in the absence of an encoded movement protein" by Ying et al reported their findings on umbravirus-like RNA viruses (ULVs) that do not encode movement protein (MP) , but could systemically infect Nicotiana benthamiana in a phloem-limited fashion when delivered by agroinfiltration. The authors generated full-length clones of CY1 and CY2 and examined their abilities to systemically infect the model plant N. benthamiana. CY1 encodes replication proteins only, but CY2 closely related CY1, which encodes an additional protein (ORF5) that has characteristics more similar to luteoviruses/poleroviruses/sobemovirus capsid proteins than to 30K MPs and ORF5 was dispensable for infection of CY2, but was associated with faster symptom development. In systemically infected CY2-infected plants, small virus-like particles (VLPs) were identified, suggesting that CY2 uses ORF5 as a CP, which would explain the absence of an umbravirus-type helper virus in a sample from hemp containing CY2.

When CY1 was used as a virus-induced gene silencing (VIGS) vector to reduce PP2 transcripts, a phloem protein 2 (PP2), an enigmatic, dimeric, chitin-binding lectin encoded by a large gene family is highly abundant in phloem sap and is considered to be a non-specific RNA-binding protein, in which CY1 accumulation was reduced in systemic leaves, supporting the possibility that PP2 is the MP for CY1.

The story is interesting and the paper is well written. Before considering for acceptance publication, there are some points below that need to be addressed and corrected before

Major concerns:

1. Does reducing PP2 expression also affects CY2 accumulation and systemic movement?

Minors:

1. Line 39 and 145: it should be clear which protein from CY1 interacts with PP2.

Rev. 2:

Ying et al. report the first plant virus (CY) that establishes a systemic infection with no need of a movement protein (MP). This finding challenges the dogma that currently known plant viruses need of this protein to colonize their host. Therefore, this work represents a ground-breaking advance in our understanding of plant-virus interactions. Owing to the relevance of the subject and conclusions of the manuscript, the authors not only demonstrate that CY1, which only encodes replication proteins, systemically colonizes different hosts, but also demonstrate that CY2, which encodes a protein potentially acting as a MP (ORF5) do the same even in the absence of ORF5, which is more likely a coat protein. Finally, the authors provide an explanation on how these viruses manage to establish systemic infections by showing that they appear to interact with host proteins with similar activities than viral MPs such as PP2. The manuscript is very well written, experiments are presented in a logical manner and with a clear connection and rationale linking them, and results support the conclusions of the paper. Overall, I find this to be a notable contribution to PLoS Biology and I only have some comments on aspects that need to be clarify but won´t change the conclusions of the work. In order of appearance:

222-225. Table 1. I had some difficulties to understand the statistical analysis here. It seems that numbers reflect percentages of symptomatic plants over 45-50 individuals assayed. Then, how did the authors perform a Student´s t-test? What was considered a replicate here? Would not be more logical just to apply a Fisher exact test, which is more appropriate for comparing frequencies? Conclusions are unlikely to change, but I would much appreciate if the authors could clarify this point.

228-235 and Figure 4. The authors state that CY2sgm1 and 2 mutants do not express the subgenomic RNA encoding ORF5 and this is supported by data on Figure 4B. However, I am intrigued by data on Figure 4C which, in the middle panel, shows a band of seemingly the same size of the subgenomic RNA for lines of single infections by CY2sgm1. Is it possible that small quantities of subgenomic RNA would be expressing?

333-342. Detection of VLPs point to ORF5 as a CP involved in virion formation. However, it might be well possible that these are empty virions that can self-assemble as has been shown for other plant viruses. I understand that demonstrating that the detected VLPs are infectious/transmissible is out of the scope of the work. However, did the authors at least try to purify these particles and to check if viral RNA could be detected by disassembling them?

390-403 and Figure 8. If I understood correctly, the random hairpin acts as a negative control both to check that only the PP2 hairpin affects PP2 expression and that introducing the hairpin does not affect the outcome of infection as compared with the WT. Although the statistical test is not shown, looking at figure 8 I assume that CY1-WT and CY1-random induce similar levels of PP2 expression as expected. Thus, one would expect to see similar levels of virus accumulation in WT and random CY1s. However, panel C shows that CY1-random accumulates at higher levels than the WT. This could suggest that the random hairpin is somehow affecting the progress of the infection. Did the authors consider this possibility?

On the same figure, in panel B most replicates of CY1-random show higher PP2 expression that replicates of CY1-PP2. Oddly, in panel C there is at least one CY1-random replicate in which virus accumulation is 0. Does this mean that PP2 can be over-expressed even in the absence of the virus? How this would affect the authors conclusions?

Finally, and I apologize for repeating myself, it is unclear why in these analyses a different statistical test is used. First, the LSD (which is a post-hoc test) is presented before the main test, which I understand is the Omnibus test. Second, why using here the omnibus test instead the Student´s t-test? Perhaps it would be worth adding a section on the Material and Methods explaining the statistical methodology.

461. In several parts of the manuscript a defective RNA is used and mentioned to be described elsewhere, but I found no description of it in the text. If the authors meant that it will be described in a different article, perhaps it would be worth asking if the analyses using the defective RNA are central to make their point, and if the answer is "no" I would recommend removing them. If the authors chose to maintain the analyses with the defective RNA, I think it would be necessary to add at least a brief description of it to facilitate the reader to understand the results.

490-495. I had some problems following this part of the text. Do the authors mean that part of the CY2 clone was generated using CY1 as a template because the CY2 sequence was unknown? Would not this make a chimera? To which extent could this affect the authors conclusions?

As a general minor comment, I suggest the authors to revise scientific names of plant species as they are not consistently written in italics.

# Author comment: Author response to Decision Letter 1

**DOI:** 10.1371/journal.pbio.3002600.r003

28 Feb 2024

Attachment Submitted filename: Response to reviewers.pdf

# Editor report: Decision Letter 2

**DOI:** 10.1371/journal.pbio.3002600.r004

**Authors:** Ines Alvarez-Garcia

12 Mar 2024

Dear Dr Simon,

Thank you for your patience while we considered your revised manuscript entitled "Independent systemic infection of umbravirus-like RNA viruses in the absence of an encoded movement protein" for publication as a Research Article at PLOS Biology. This revised version of your manuscript has been evaluated by the PLOS Biology editors and the Academic Editor.

Based on our Academic Editor's assessment of your revision, we are likely to accept this manuscript for publication, provided you satisfactorily address the policy-related requests stated below.

In addition, we would like you to consider a suggestion to improve the title:

"Umbravirus-like RNA viruses are capable of systemic plant infection in the absence of movement proteins"

As you address these items, please take this last chance to review your reference list to ensure that it is complete and correct. If you have cited papers that have been retracted, please include the rationale for doing so in the manuscript text, or remove these references and replace them with relevant current references. Any changes to the reference list should be mentioned in the cover letter that accompanies your revised manuscript.

We expect to receive your revised manuscript within two weeks. 

To submit your revision, please go to https://www.editorialmanager.com/pbiology/ and log in as an Author. Click the link labelled 'Submissions Needing Revision' to find your submission record. Your revised submission must include the following:

- a cover letter that should detail your responses to any editorial requests, if applicable, and whether changes have been made to the reference list

- a Response to Reviewers file that provides a detailed response to the reviewers' comments (if applicable, if not applicable please do not delete your existing 'Response to Reviewers' file.)

- a track-changes file indicating any changes that you have made to the manuscript. 

NOTE: If Supporting Information files are included with your article, note that these are not copyedited and will be published as they are submitted. Please ensure that these files are legible and of high quality (at least 300 dpi) in an easily accessible file format. For this reason, please be aware that any references listed in an SI file will not be indexed. For more information, see our Supporting Information guidelines:

https://journals.plos.org/plosbiology/s/supporting-information

*Published Peer Review History*

Please note that you may have the opportunity to make the peer review history publicly available. The record will include editor decision letters (with reviews) and your responses to reviewer comments. If eligible, we will contact you to opt in or out. Please see here for more details:

https://blogs.plos.org/plos/2019/05/plos-journals-now-open-for-published-peer-review/

*Press*

Should you, your institution's press office or the journal office choose to press release your paper, please ensure you have opted out of Early Article Posting on the submission form. We ask that you notify us as soon as possible if you or your institution is planning to press release the article.

*Protocols deposition*

To enhance the reproducibility of your results, we recommend that if applicable you deposit your laboratory protocols in protocols.io, where a protocol can be assigned its own identifier (DOI) such that it can be cited independently in the future. Additionally, PLOS ONE offers an option for publishing peer-reviewed Lab Protocol articles, which describe protocols hosted on protocols.io. Read more information on sharing protocols at https://plos.org/protocols?utm_medium=editorial-email&utm_source=authorletters&utm_campaign=protocols

Please do not hesitate to contact me should you have any questions.

Sincerely,

Ines

--

Ines Alvarez-Garcia, PhD

Senior Editor

PLOS Biology

ialvarez-garcia@plos.org

------------------------------------------------------------------------

DATA POLICY: IMPORTANT - PLEASE READ

You may be aware of the PLOS Data Policy, which requires that all data be made available without restriction: http://journals.plos.org/plosbiology/s/data-availability. For more information, please also see this editorial: http://dx.doi.org/10.1371/journal.pbio.1001797

Note that we do not require all raw data. Rather, we ask that all individual quantitative observations that underlie the data summarized in the figures and results of your paper be made available in one of the following forms:

1) Supplementary files (e.g., excel). Please ensure that all data files are uploaded as 'Supporting Information' and are invariably referred to (in the manuscript, figure legends, and the Description field when uploading your files) using the following format verbatim: S1 Data, S2 Data, etc. Multiple panels of a single or even several figures can be included as multiple sheets in one excel file that is saved using exactly the following convention: S1_Data.xlsx (using an underscore).

2) Deposition in a publicly available repository. Please also provide the accession code or a reviewer link so that we may view your data before publication. 

Regardless of the method selected, please ensure that you provide the individual numerical values that underlie the summary data displayed in the following figure panels as they are essential for readers to assess your analysis and to reproduce it:

Fig. 8B, C

NOTE: the numerical data provided should include all replicates AND the way in which the plotted mean and errors were derived (it should not present only the mean/average values).

Please also ensure that figure legends in your manuscript include information on WHERE THE UNDERLYING DATA CAN BE FOUND, and ensure your supplemental data file/s has a legend.

Please ensure that your Data Statement in the submission system accurately describes where your data can be found.

------------------------------------------------------------------------

BLOT AND GEL REPORTING REQUIREMENTS:

Many thanks for providing the original, uncropped gels. I have checked the files and I am missing the original images for the following files, thus please upload a new file including these:

Fig. S2B, Fig. S4 and Fig. S8B, C 

------------------------------------------------------------------------

DATA NOT SHOWN or UNPUBLISHED

- Please note that per journal policy, we do not allow the mention of "data not shown", "personal communication", "manuscript in preparation" or other references to data that is not publicly available or contained within this manuscript. Please either remove the mention of these data in line 419 or provide figures presenting the results.

# Author comment: Author response to Decision Letter 2

**DOI:** 10.1371/journal.pbio.3002600.r005

14 Mar 2024

Attachment Submitted filename: Second response to the editor.pdf

# Editor report: Decision Letter 3

**DOI:** 10.1371/journal.pbio.3002600.r006

**Authors:** Ines Alvarez-Garcia

26 Mar 2024

Dear Dr Simon,

Thank you for the submission of your revised Research Article entitled "Umbravirus-like RNA viruses are capable of independent systemic plant infection in the absence of encoded movement proteins" for publication in PLOS Biology. On behalf of my colleagues and the Academic Editor, Wenbo Ma, I am delighted to let you know that we can in principle accept your manuscript for publication, provided you address any remaining formatting and reporting issues. These will be detailed in an email you should receive within 2-3 business days from our colleagues in the journal operations team; no action is required from you until then. Please note that we will not be able to formally accept your manuscript and schedule it for publication until you have completed any requested changes.

Please take a minute to log into Editorial Manager at http://www.editorialmanager.com/pbiology/, click the "Update My Information" link at the top of the page, and update your user information to ensure an efficient production process.

PRESS

We frequently collaborate with press offices. If your institution or institutions have a press office, please notify them about your upcoming paper at this point, to enable them to help maximise its impact. If the press office is planning to promote your findings, we would be grateful if they could coordinate with biologypress@plos.org. If you have previously opted in to the early version process, we ask that you notify us immediately of any press plans so that we may opt out on your behalf.

We also ask that you take this opportunity to read our Embargo Policy regarding the discussion, promotion and media coverage of work that is yet to be published by PLOS. As your manuscript is not yet published, it is bound by the conditions of our Embargo Policy. Please be aware that this policy is in place both to ensure that any press coverage of your article is fully substantiated and to provide a direct link between such coverage and the published work. For full details of our Embargo Policy, please visit http://www.plos.org/about/media-inquiries/embargo-policy/.

Many congratulation and thanks again for choosing PLOS Biology for publication and supporting Open Access publishing. We look forward to publishing your study. 

Sincerely, 

Ines

--

Ines Alvarez-Garcia, PhD

Senior Editor

PLOS Biology

ialvarez-garcia@plos.org
